# Effect of mineralogical variations on physico-mechanical and thermal properties of granitic rocks

**DOI:** 10.1038/s41598-023-36459-9

**Published:** 2023-06-26

**Authors:** El Saeed R. Lasheen, Mohammed A. Rashwan, Mokhles K. Azer

**Affiliations:** 1 Geology Department, Faculty of Science, Al-Azhar University, P.O. 11884, Cairo, Egypt; 2 Geological Sciences Department, National Research Centre, 33 El Bohooth st. (former El Tahrir st), Dokki, P.O. 12622, Giza, Egypt

**Keywords:** Mineralogy, Engineering

## Abstract

The present study aims to explain the geochemical and mineralogical details of the granitic rock types in Gabal EL-Faliq area, South Eastern Desert of Egypt, in relation to geotechnical engineering and their suitability as dimension stones. The objective of the current research was achieved through two steps; the first step involved geological studies such as the petrographic, geochemical, and mineralogical investigations. The second and applicable step involved the geotechnical assessment of the studied rocks by measuring their engineering properties such as physical, mechanical, and thermal expansion properties. The petrographic investigation revealed that the studied granitic rocks are divided into two main classes: (1) gneissose granites (Biotite–Perthite) of medium to fine-grained size and (2) alkali-feldspar granites of coarse to medium-grained size. Mineralogically, the studied rocks are composed mainly of albite, orthoclase and quartz in varying proportions, along with some accessory minerals such as apatite and rutile in addition to some minor quantities of iron-group minerals such as hematite and ilmenite. The engineering properties showed that the maximum water absorption and apparent porosity values are 0.34% and 0.77%, respectively, while the minimum bulk density is 2604.03 kg/m^3^. The compressive strength ranges from 999.68 to 2469.10 kg/cm^2^, while the abrasion resistance varies from 29.67 to 54.64 Ha. The increase in albite content led to an increase in water absorption while a decrease in bulk density and compressive strength. The increase in the grain size led to an increase in apparent porosity and a decrease in mechanical properties. A Great variation in the expansion coefficient as well as the length change, occurs under changes in temperature, mineral composition, and physical properties. The increase in heating temperatures caused an insignificant increase in linear thermal expansion with a maximum value of 0.0385% at 100 °C. These results indicated the suitability of the studied granites as dimension stones for use in indoor and outdoor decorative purposes (cladding/paving) under variable temperature conditions.

## Introduction

Dimension stones are any type of natural stones or rock products, excluding all manmade materials that simulate stone, that can be cut to obtain elements having well-determined geometrical shapes or sizes and satisfy the normal requirements of polishing ability, color, texture, and surface finish to be utilized as building and ornamental materials such as building facing, paving stone, curbing, monuments and memorials, and other industrial products^1–5^. Therefore, the classification of natural stones as dimensional stones is governed by their appearance and dimensions which are the two main criteria. Furthermore, the dimension stones should satisfy the strength, polish-ability and resistance to physical and chemical weathering^6^.

The igneous, metamorphic, and sedimentary rocks are the three main categories of natural stones based on origin and are widely used all over the world in many applications such as dimension stones due to the great variety of their appearances in addition to their high compactness and durability that enabled them to be used in flooring, cladding, paving, funeral monuments, and statues^7^. Natural stones are the most widely applied material in ancient heritage constructions such as pyramids, castles, and palaces. The use of dimension stones in traditional constructions is closely related to the distribution of rock outcrops^8^.

According to (ASTM C119)^1^, the dimension stones were classified into several groups including granite, limestone, marble, quartz-based (quartzite), and slate groups in addition to other dimension stones such as alabaster and serpentine. However, the two most common groups of natural dimension stones are marble or calcareous material groups and granite or siliceous material groups in addition to other stone types like quartzite and slate^3,4^. The first group (marble) comprises the whole class of carbonate rocks, composed normally of calcite and dolomite and capable of taking sawing and polishing, outside the limits of the mere lithologic characterization^3,9^. It is ranging in composition from stones of pure carbonate to that containing very little carbonate that is classed commercially as marble-like (serpentine marble)^1^. The second common group (granite) comprises, in a commercial definition, the whole set of eruptive or igneous rocks having granular structure and poly-mineral composition, irrespective of the content of quartz^3^.

The production of dimension stones is increasing rapidly as a result of the massive expansion of building construction projects due to the continuous increase in the population. The worldwide net production of dimension stones reached about one hundred fifty million tons in 2017 by 27 countries^10^, with a major sharing of about 72% by China, India, Turkey, Iran, and Italy^6,11^.

Locally, Egypt is distinguished by a wide distribution of different types of natural stones of more than fifty brands that can be utilized as dimension and ornamental stones^12,13^. Therefore, Egypt occupied the seventh place in the world in the production of dimension stones^14^ with a production volume of about 5.25 Million tons with 4% global sharing as mentioned in Montani Report 2018^10^.

In Egypt, the granitic intrusions constitute about 60% of the total Neoproterozoic rocks^15,16^. They are attributed to syn-orogenic calc-alkaline, late- post-orogenic highly fractionated granitoids and post-orogenic alkaline granitic rocks^7,17^. Furthermore, these intrusions incorporate grey, white, pink, and red granites^18^. The generation of such granitic rocks was proposed by two possible mechanisms: the first mechanism is magma differentiation (fractional crystallization or assimilation) of mantle-derived basaltic melt, while the second proposed mechanism is partial melting of crustal rocks (mafic-acidic igneous or sedimentary rocks). The granitic rocks attracted the attention of many authors due to their wide exposure and composition, well appearance, and economically hosting of significant rare earth metals such as Nb, Ta, U, Th, Zr, Sn, and W^7,19–21^.

According to (Alzahrani et al. 2022)^7^, the variation in the mineralogical and chemical composition of the granitic rocks resulted in a variation in their thermal expansion and spectral reflectance behaviors in addition to their physical and mechanical properties. They stated that the low iron oxide granitic rocks have a high spectral reflection in the (VIS-NIR) and (SWIR) spectral regions. Moreover, the granitic rocks of high iron and/or low quartz content revealed a high physical and mechanical performance.

According to (Siegesmund et al. 2018)^22^, thermal expansion is a physical property, which occurs under the change in all material's temperatures, where most of the materials upon heating and cooling expand and contract; respectively. In thermally sensitive materials, temperature changes cause a pronounced alteration that is well known as physical or mechanical weathering. Though the relatively small extent of thermal expansion with its minor effects on the volume change and bulk density of the rocks, the variations in the characteristics of thermal expansion of different minerals in the assemblage of mineral grains can cause structural damage upon heating the rock. One of the causes of rock deterioration is thermal expansion. This results from that the heat conductivity of granitic rocks is bad, and as thermal action on the rock surface is more intense than in its interior, tensions develop causing the formation of cracks in the outer surface of the rock^23^.

The thermal studies on granitic rocks collected from the Himalayan regions and the Peninsular shield in India revealed that the mineral composition, grain orientation, crack porosity, rate of heating, and thermal cycling have a great effect on their linear thermal expansion coefficient of granites^24^.

Plevova et al. (2016)^25^ applied several thermal (DTA, TG, TMA, α_H_), mineralogical, and petrographical investigations on six granitic rock samples from three countries. They observed a relative similarity in the thermal expansion values, while a little difference in the shapes of their TMA curves. They attributed such differences to their quartz and feldspar content, feldspar crystallinity and the ratio of anorthite and albite in the granite rocks.

The variations in the thermal expansion coefficients of the individual rock-forming minerals as well as the rock fabric are considered the important parameters that the thermal expansion of a rock is dependent on Ref.^22^. They investigated the effect of temperature (up to 120 °C) as well as the mineralogical composition on the thermal properties of several granitoid rocks. They observed much higher thermal expansion coefficients in the granitoid rocks rich in quartz compared to that rich in K-feldspar or plagioclase content. However, the contribution of biotite and hornblende minerals modifies the thermal expansion of whole rock.

There are several factors influencing the degree of thermal expansion in rocks such as their mineralogical composition (especially the quartz and calcite content), crystal orientation (structure) in addition to their degree of porosity^23^. They determined the linear thermal expansion coefficients of some Brazilian granitic rocks commercially used as building facings within a temperature range of 0 to 50 °C and compared these values with the quartz content, apparent porosity, and grain size of the studied rocks. They observed that the increase in quartz content of the granitic rocks led to an increase in their thermal coefficient values. On the other hand, a decrease in thermal coefficient was observed with increasing apparent porosity and grain size.

Mineral constituents, grain size, texture, and degree of alteration of the rocks, are the main factors affecting the engineering properties and durability performance or the rate of degradability of stones^26–32^. Hemmati et al. (2020)^29^ studied the effect of mineralogy and texture of different crystalline igneous rocks on their strength properties and found a relationship between the compressive and tensile strength of the studied igneous rocks and their quartz/feldspar size ratio. The effect of temperature changes on the physical and mechanical properties of dimension stones, especially igneous rocks, is highly obvious due to their wide mineralogical composition that exhibits a variable thermal variation under different temperature degrees^33^.

Other studies have dealt with the petrographic characteristics of stone aggregates such as alteration degree, mineral composition, and stone texture and their effects on the durability performances of concrete^34,35^. They found that the final strength of the concrete specimens was affected by mineralogy and microstructure of the coarse aggregates.

### Study significance

Despite the abundance of intrusive igneous rocks in the Gebel El-Faliq area, South Eastern Desert of Egypt with great variations in their colors and types, there are no studies were carried out on the granitic rocks of this area related to their suitability as dimension stones. Moreover, the global climatic changes accompanied by the increase in temperatures may have a great impact on the engineering properties of these rocks. Therefore, the main objective of the current research was directed to evaluate the thermal behavior and some engineering properties of different varieties of the Neoproterozoic granitic rocks located in Gebel El-Faliq area, South Eastern Desert, Egypt, and their suitability as dimension stones for use in the cladding and flooring applications. Moreover, the influence of the mineralogical and chemical compositions of the different granitic rocks on their thermal and engineering properties was also studied. Additionally, the results of engineering properties of the studied granitic rocks were compared to the international standard specifications related to ornamental (dimension) stones. To achieve the above-mentioned objectives, several analyses and investigations were carried out on the collected rock types such as elementary analysis by X-ray fluorescence (XRF), petrographical examination by polarizing light microscope, thermal expansion coefficient by dilatometer, in addition to measuring some engineering properties such as physical properties (apparent porosity, water absorption, and bulk density) and mechanical properties (compressive strength, abrasion resistance) according to the international standard test methods (ASTM).

## Geological background

**Figure 1:**
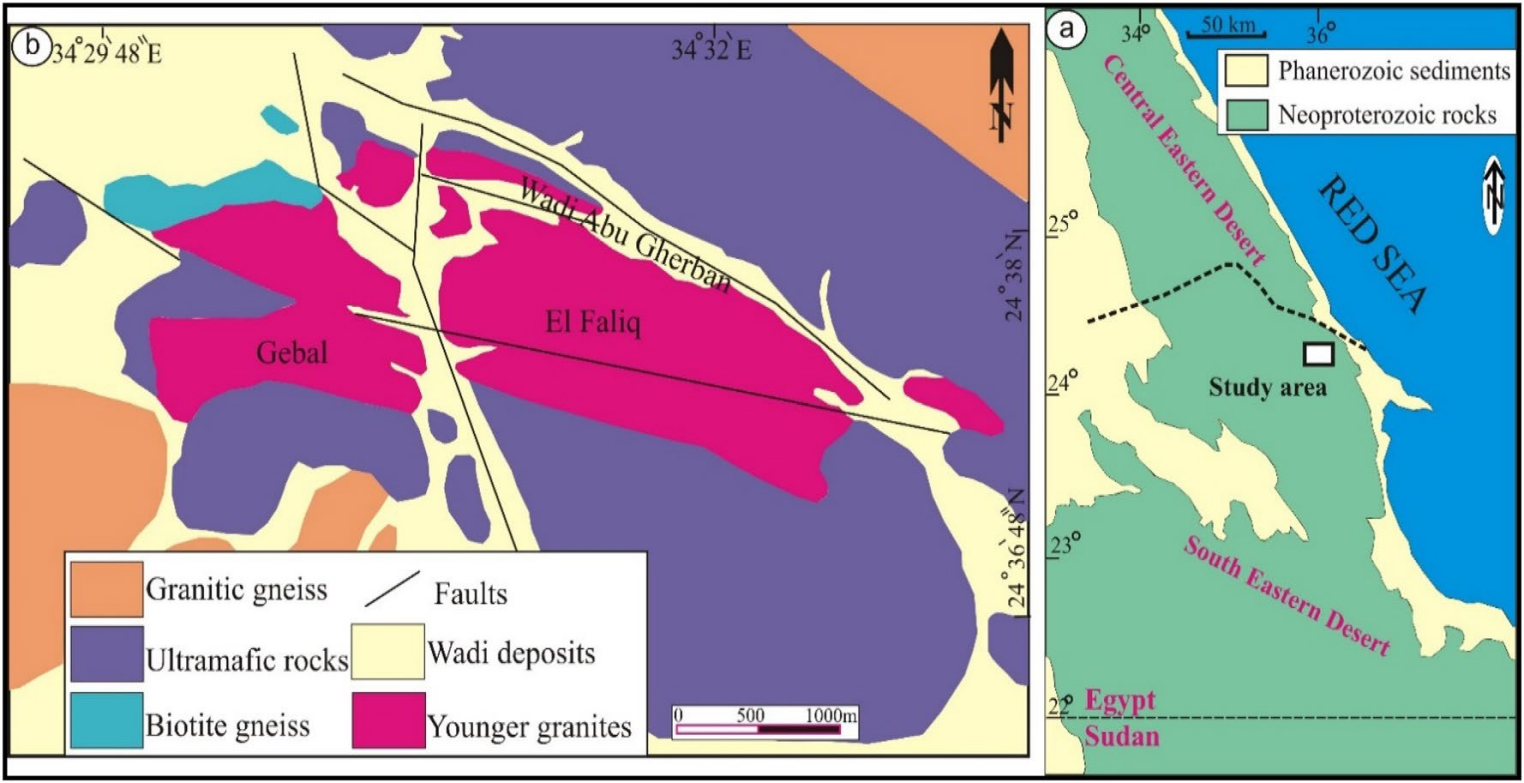
Geologic map of Gabal El Faliq area (modified after: Mahmoud 2019^44^; Saleh et al. 2014^45^).

Egyptian Neoproterozoic rocks are well exposed parallel to the Red Sea (Eastern Desert), South Sinai and Uwainate area, Northern territory of the Arabian Nubian Shield (ANS). ANS represents well juvenile crust, which is formed by arc accretion, followed by crustal thickening and closing of Mozambique Ocean^17,36–41^. Evolution of ANS is involved by large granitic intrusions with a wide diversity in mineralogical and tectonic regime^7,17,20^. Gebal El Faliq is delineated by Latitude 24° 36' and 24° 37' N and Longitude 34° 28' and 34° 33' E covering ~10 km^2^ (Fig. 1). It locates in southwestern of Wadi Ghadir-Hafafit, South Eastern Desert, Egypt. Mylonitic, ophiolitic ultramafic rocks, younger granites and post-granitic dikes and pegmatites are the main rocks crop out in the investigated area. Low relief of mylonitic rocks is exposed in the northwestern side of Gabal El Faliq younger granites. Ophiolitic rocks, as they are widely distributed in the Eastern Desert (Central and Southern sides)^17,42,43^, they cover most of the examined area, which are represented by dunite (or peridotites)^44^ and mélange rocks^45^. Gabal El Faliq is represented by younger granitic rocks with elongated sheets along NW-SE trend certainly along Wadi Abu Gherban. They reveal low- to moderate-topographic (664 m above sea level) relief that is represented by monzogranites and alkali feldspar granites^45^. They are dissected by numerous faults (sinistral-strike slip fault as a major fault), therefore, sheared and gneissose texture are well exposed, particularly along shear zone and fault planes. They are injected by basic dykes and pegmatites with the main NW-SE direction. Some of these pegmatites with variable widths and lengths are enriched with rare metals, such as Zr, Nb, Ta, Th, U and REE^44^. In addition, they possess xenoliths from the surrounding rocks certainly along their margins. Some alteration features are observed such as hematitization and kaolinization, particularly along shear zone and fault planes.

According to (Global Carbon Project, 2021), Egypt accounts for only 0.6 percent of annual global carbon dioxide (CO_2_) emissions, however, it is becoming one of the most heavily affected by extreme weather patterns^46^. Eid et al.^47^ analyzed the monthly, seasonal and annual values of temperature over Egypt during the period 1960-2016. They found that the differences between the values of mean temperature in the north and the south of Egypt are measured at 5 °C, 8 °C, 9 °C, and 6 °C in winter, spring, summer, and autumn; respectively. They found that the difference between the values of mean annual temperature in the north and the south of Egypt is about 7.5 °C.

Regarding the total annual precipitation, Nashwan and Shahid^48^ gives a future look at the changes in precipitation amount and characteristics over Egypt by the end of the century. They revealed an increase in annual precipitation by up to 54% mostly in the north, with a decrease in winter precipitation by 35% Markus et al.^49^ stated that the most frequently used climate classification map is that of Wladimir Köppen, presented in its latest version 1961 by Rudolf Geiger. It is used to denote different climate regions on Earth based on local vegetation.

## Materials

**Figure 2:**
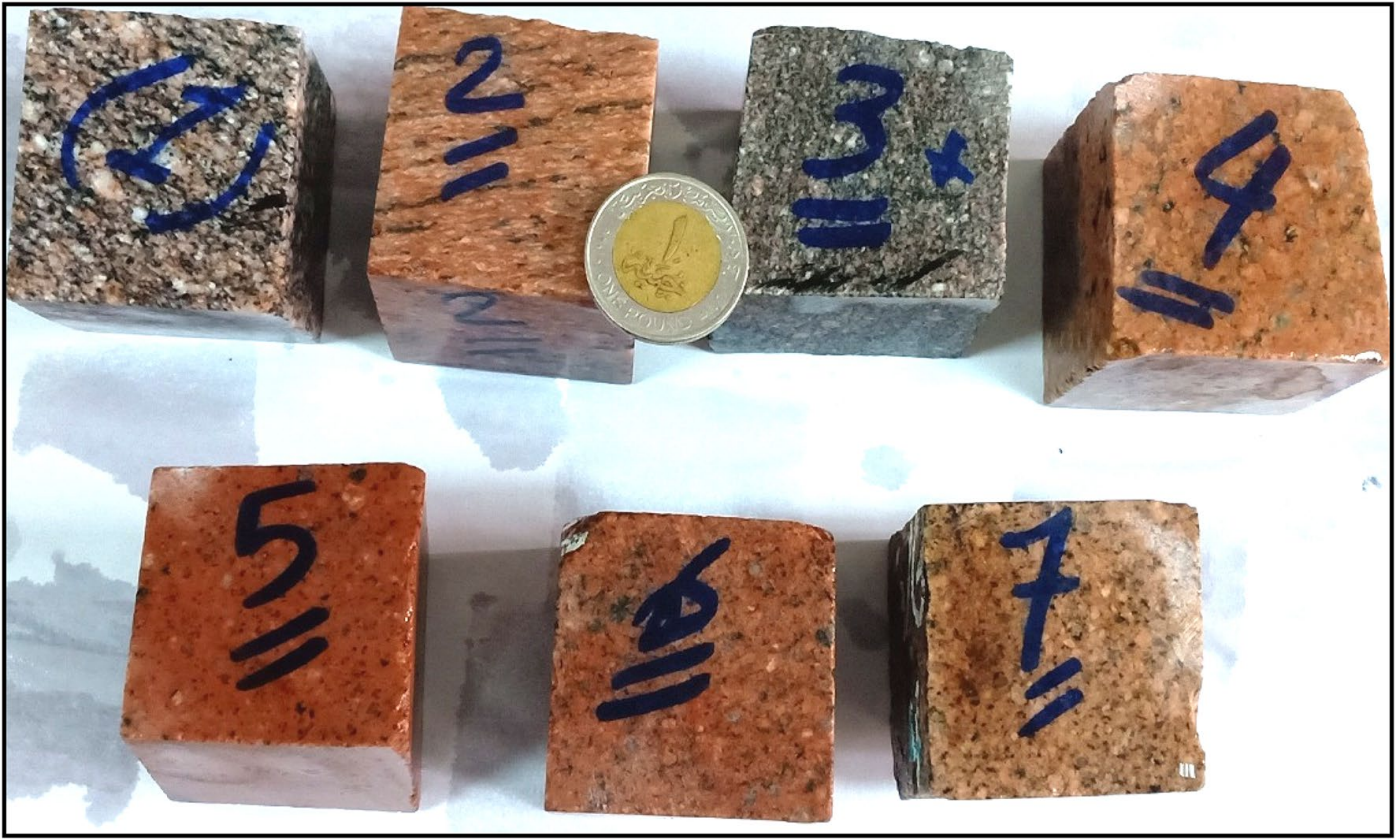
Photographs showing hand specimens of different types of Gabal El Faliq granitic rocks.

**Figure 3:**
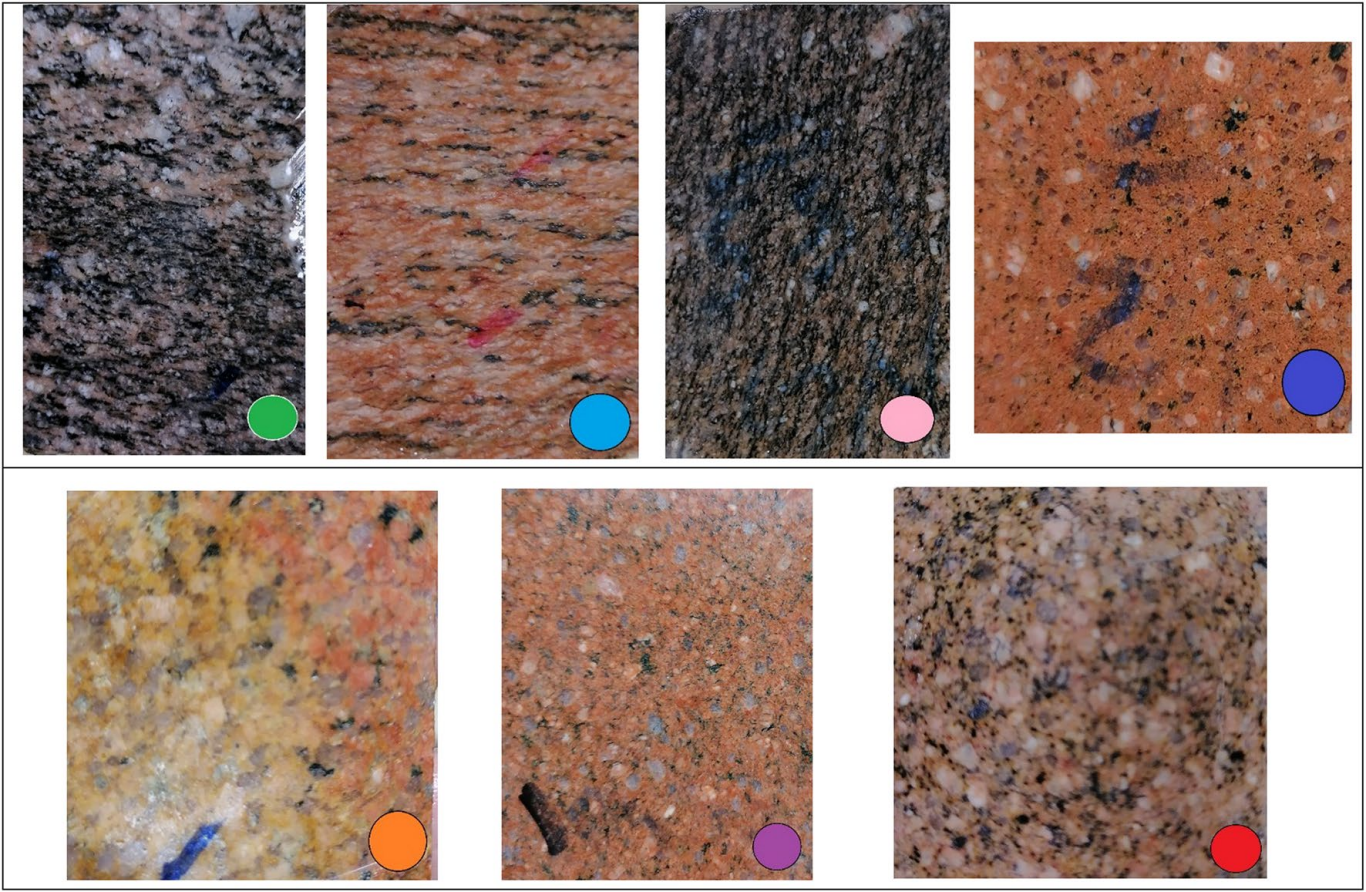
Photographs showing polished surface of selected types of granitic rocks.

For the current research, seven intrusive igneous rocks, of a younger granitic type, were collected from Gabal El-Faliq study area as shown in (Fig. 2). The difference in rock types sampling was based on the hand specimen's variation in color (due to variation in mineral composition) and texture (due to variation in grain size and orientation). Some representative samples were polished for visual illustration as shown in (Fig. 3) Regarding samples preparation, each granitic rock type was divided into three shapes as follows: two cylindrical specimens with dimensions of 20 mm length and 5 mm diameter, six cubic specimens with dimensions of 50 x 50 x 50 mm, and three specimens with dimensions of 50 mm square and 25 mm thickness. The gneissose rock samples were cut perpendicular to their grain orientation.

## Methods

### Elementary analysis and normative mineral composition

The collected granitic rocks of different types were configurated for elementary analysis through grinding and sieving under 74 µm sieve size and then dried in an oven dryer at 105 ± 5 °C overnight for complete dryness. Part of each prepared sample was ignited at 1000 °C for measuring the loss on ignition (L.O.I) % following the standard test method (**ASTM E-1621**). The major and trace elements of the prepared rock samples were measured at the National Research Center (NRC), using X-Ray Fluorescence (XRF), Axios, PANalytical 2005 with Sequential WD-XRF Spectrometer. Based on the XRF analysis, the normative mineral composition of the studied granitic rocks was calculated using (CIPW normative method) according to (Streckeisen 1976)^50^.

### Petrographic examination

For identifying the rock-forming minerals, grain texture, and alteration or deformation indices using a polarized light microscope (petrographic analysis), thin sections of the rock types were prepared by cutting into slabs of few millimeters thick using rock cutting machine with a diamond saw, polished using rotary grinding machine and then mounted on a glass slide using Canada Balsam and smoothly grounded using progressively finer abrasive grit until the sample reaching a thin slice of approximately 0.03 mm thick.

### Engineering properties

The studied granitic rocks were geotechnically evaluated in terms of 1) physical properties such as water absorption, apparent porosity, and bulk density, 2) mechanical properties such as compressive strength and abrasion resistance according to (ASTM C615)^51^. The samples were cut using a rock-cutting machine into two dimensions as follows: (a) 50 × 50 × 50 mm for measuring water absorption and bulk density according to (ASTM C97)^52^ and compressive strength according to (ASTM C170)^53^. (b) 50 × 50 × 25 mm for measuring the abrasion resistance according to (ASTM C241)^54^. The rock samples were cut to sizes and their physical and mechanical properties were measured in the marble and granite testing lab (MGTL) at National Research Centre (NRC).

The water absorption, apparent density, dry bulk density, and saturated-surface dry bulk density were calculated to the following equations:

1 $${\text{Water absorption}},\,\% \, = \,{1}00\,*\,\left( {{\text{SSD}}\,{\text{weight }}{-}{\text{ Dry}}\,{\text{weight}}} \right)\,/\,{\text{Dry}}\,{\text{weight,}}$$

2 $${\text{Apparent porosity}},\,\% \, = \,{1}00\,*\,\left( {{\text{SSD}}\,{\text{weight}}\,{-}\,{\text{Dry}}\,{\text{weight}}} \right)\,/\,\left( {{\text{SSD}}\,{\text{weight}}\,{-}\,{\text{Suspended}}\,{\text{weight}}} \right),$$

3 $${\text{Dry}}\,{\text{Bulk density}},{\text{ kg}}/{\text{m}}^{{3}} = { 1}000 \, *{\text{ Dry weight}}\,/\,\left( {{\text{SSD weight }}{-}{\text{ Suspended weight}}} \right),$$

4 $${\text{SSD Bulk density}},{\text{ kg}}/{\text{m}}^{{3}} = { 1}000 \, *{\text{ SSD weight}}\,/\,\left( {{\text{SSD weight }}{-}{\text{ Suspended weight}}} \right).$$

The compressive strength is calculated according to the equation:

5 $${\text{Compressive strength}},{\text{ MPa }} = {\text{ Total load }}\left( {\text{N}} \right) \, /{\text{ Loading area }}\left( {{\text{mm}}^{{2}} } \right).$$

### Thermal studies

The cylindrically-shaped rock samples of around 20 mm in length and 5 mm in diameter were used for measuring linear thermal expansion coefficients (***α***) and length change (thermal strain) (***dL/Lo***) by stepwise heating at a rate of 5 °C / min up to 1000 °C using a Dilatometer instrument (NETZSCH DIL 402 PC model). The test was carried out in the central lab of the institute at the National Research Centre (NRC).

### Ethical approval

This article does not contain any studies with human participants or animals performed by any of the authors.

### Consent to participate

All authors are agreed to be listed as authors in current version of manuscript.

## Results and discussions

### Petrographic investigation

The petrographic examination of Gabal El Faliq granitic rocks was performed using a polarizing microscope to identify their mineralogical constituntes and textural features to revels some significant attributes affected these rocks. Based on the mineralogical composition and the main textural relationship, Gabal El Faliq granitic rocks are divided into two major types: gneissose granites and alkali feldspar granites according to IUGS classification^50^. The gneissose type (with mainly porphyritic texture and elongated crystals type), includes the rock types F1, F2, and F3, whereas the alkali feldspar type (hypidiomorphic and rarely porphyritic textures), possesses coarse-grained rock type F4 and medium-grained rock types F5, F6, and F7. All samples are investigated as below:

### Gneissose granite type (F1; Biotite, medium-grained)

**Figure 4:**
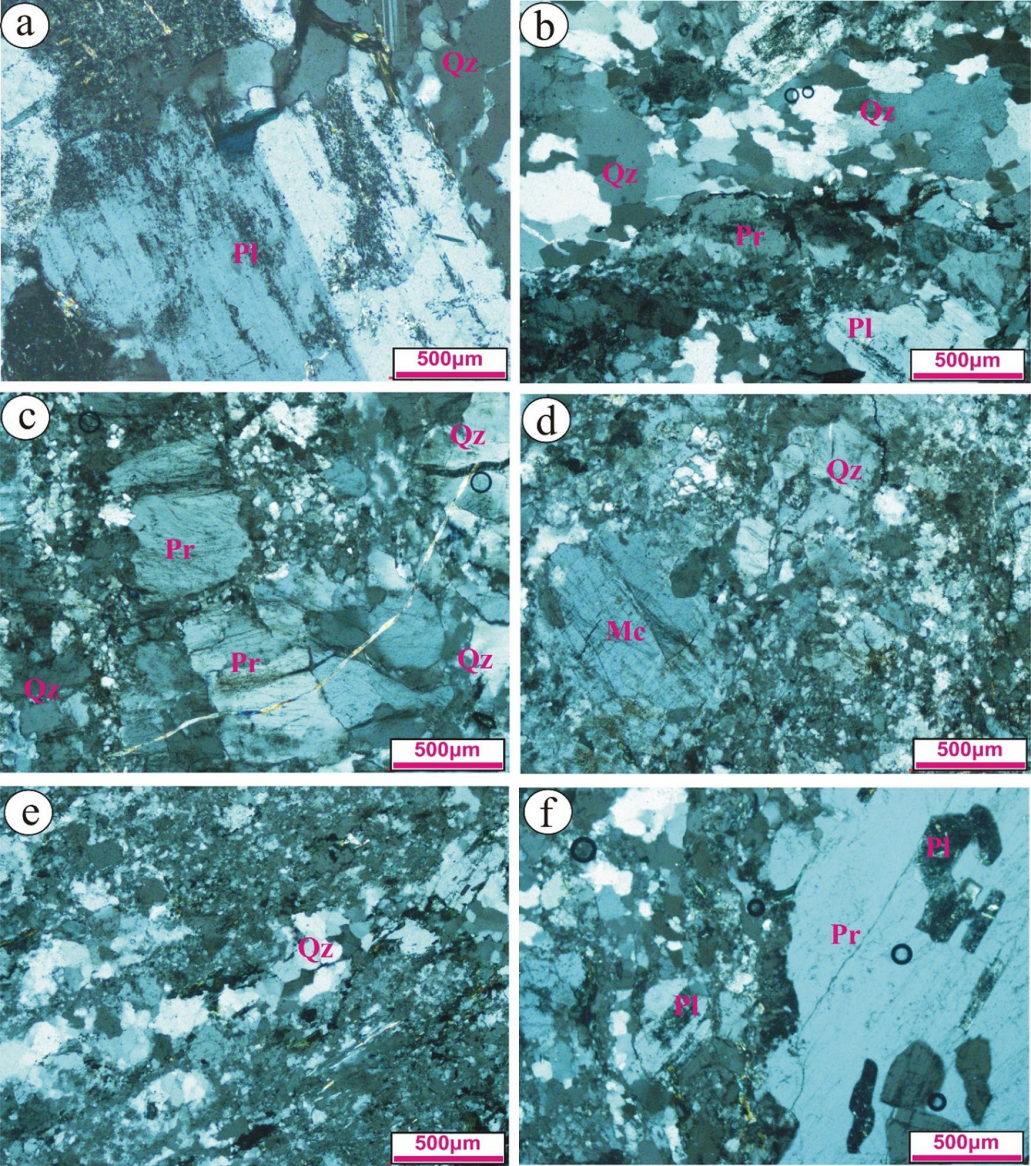
Photomicrographs reveal: (**a**) Highly saussuritized phenocrysts surrounded by fine-grained quartz and plagioclase (F1); (**b**) Elongated quartz and perthite crystals as a result of deformation processes (F1); (**c**) Highly deformed, elongated and fractured perthite phenocrysts surrounded by fin-grained quartz (F2); (**d**) Phenocrysts of perthite surrounded by fine-grained quartz and perthite forming porphyritic texture (F2); (**e**) Fine-grained of elongated quartz and feldspar crystals (F3); and (**f**) Perthite phenocrystals enclosing fine-grained of plagioclase yielding poikiolitic texture (F3).

The samples of this type are composed mainly of plagioclase, quartz, potash feldspar and minor subordinate biotite minerals. Plagioclase occurs as phenocrysts crystal mantled by fine-grained quartz and biotite forming porphyritic texture (Fig. 4a). It occurs as medium-grained crystals, reveals extensive alteration saussuritizaion and exhibits lamellar, zoned and Carlsbad twinning. It is poikilitically enclosing fine-grained chlorite. Potash feldspars are represented by perthite and rarely microcline. Perthite reveals a xenomorphic of patchy type that mostly engulfs plagioclase. Quartz range from medium- to fine-grained crystals. It shows clear undulose extension due to deformation processes. Commonly, it occurs as elongated crystals (Fig. 4b). Biotite occurs as fine-grained (shreds), flaky crystals that are partially to completely altered to chlorite. The main textures of this rock type are myrmekitic, and porphyritic textures.

### Gneissose granite type (F2; Perthite, medium-grained)

The samples of this type are medium-grained with pink color in the hand specimen. It consists mainly of potash feldspar, quartz, and minor subordinate plagioclase minerals. Potash feldspars are represented by perthite (with a clear worm-like shape) of flamy type that is mostly fractured and filled by secondary quartz (Fig. 4c). Commonly, it occurs as phenocrysts surrounded by fine-grained quartz forming a porphyritic texture. Microcline is rare and exists as fine-grained crystals (Fig. 4d). Quartz range from fine- to medium crystals with a clear wavy extension due to deformation processes. Plagioclase is rare and mostly occurs as fine-grain. It reveals extensive saussuritizaion. Allanite and zircon are the main accessory minerals. Zircon occurs as euhedral metamect crystals. Allanite occurs as zoned, tabular crystals with brown color.

### Gneissose granite type (F3; Biotite, fine-grained)

The samples of this type are composed essentially of plagioclase, quartz, potash feldspar and minor subordinate biotite minerals. Plagioclase occurs as phenocrysts crystal mantled by fine-grained quartz forming porphyritic texture. It occurs as elongated, medium-grained crystals, and exhibits lamellar, Carlsbad and zoned twinning. It is poikilitically enclosing fine-grained quartz. It reveals a turbid surface due to slightly to extensive saussuritizaion. Quartz exist as fine-grained crystals with undulose extension. Commonly, quartz is elongated forming gneissose texture (Fig. 4e). Potash feldspars are represented by perthite and microcline. Perthite exists as phenocrystals that mostly include fine-grained plagioclase (Fig. 4f). They reveal extensive a turbid surface due to kaolinitization processes. Biotite occurs as fine-grained, and elongated flaky crystals. The main textures in this rock are myrmekitic, and porphyritic textures.

### Alkali-feldspar granite type (F4; coarse-grained)

**Figure 5:**
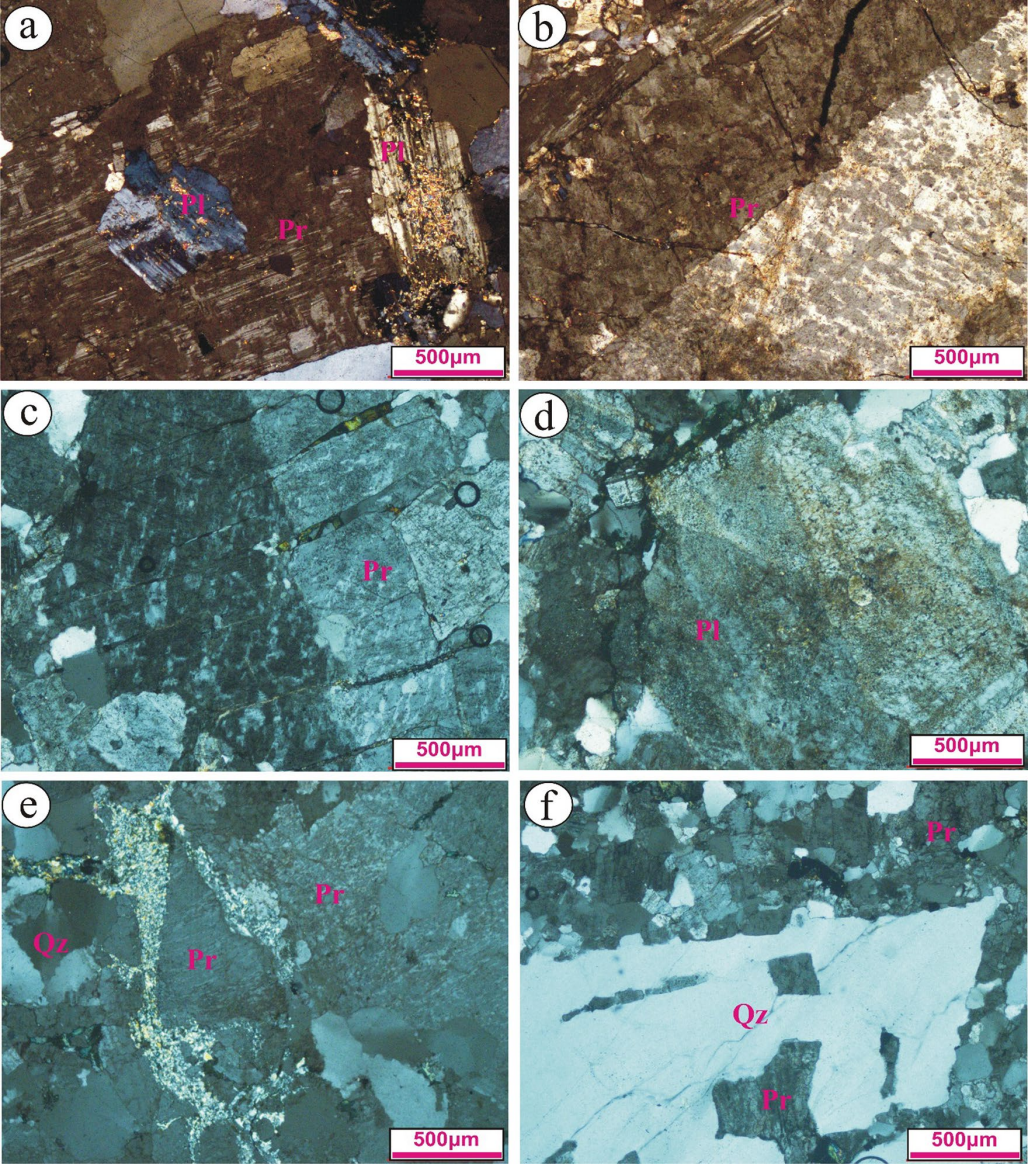
Photomicrographs reveals: (**a**) Antiperthite phenocrystals enclosing saussuritized plagioclase (F4); (**b**) Coarse-grained orthoclase perthite (F4); (**c**) Fractured coarse-grained orthoclase perthite that filled by secondary quartz (F7); (**d**) Extensive turbid surface of plagioclase crystals (F6); (**e**) Pyramid- like shape of perthite crystal surrounded by sericite (F6); and (**f**) Euhedral of skeleton quartz crystal (F5).

In this rock type, K-feldspars, quartz, plagioclase, and muscovite are the main essential minerals. Orthoclase perthite and antiperthite are the main K-feldspar minerals (Fig. 5a and b). Occasionally, it is fractured and filled by iron oxides. Occasionally, antiperthite enclosing fine-grained plagioclase minerals. Quartz exists as medium-to coarse-grained that exhibit both normal and wavy extension. Tabular crystals of plagioclase are partially to completely altered saussurite. Biotite occurs as fine-grained flaky crystals that mostly present as cluster minerals. It is altered to chlorite and stained by iron oxides certainly along its peripheries.

### Alkali-feldspar granite (F 5, 6 & 7; medium- grained)

It consists mainly of K-feldspar, quartz, and plagioclase, whereas zircon and iron oxide are the main accessory minerals. Orthoclase-perthite and microcline are the main K- feldspar minerals. They reveal a slightly turbid surface as a result of kaolinitizaion processes. Reaction rims between two perthite crystals are observed. Occasionally, orthoclase-perthite occurs as phenocrysts embedded in fine-grained quartz and plagioclase crystals (Fig. 5c). Sometimes occur as a pyramid-like shape mantled by shreds of sericite (Fig. 5e). They are fractured and filled by secondary quartz. Plagioclase occurs as prismatic crystals with clear extensive turbid surface (Fig. 5d). It exhibits pericline and lamellar twinning. Quartz reveals a wavy extension and occurs as anhedral to subhedral skeleton phenocrysts forming porphyritic texture (Fig. 5f). Biotite flaky crystals are partially altered to chlorite. High relief of euhedral zircon crystals occurred. The summary of the petrographic description of the different types of granitic rocks were presented in Table 1.

**Table 1:** Summary of petrographical description of Gebel El-Faliq granitic rocks.

Rock code	Petrographic description												
	Predominant grain size	Rock nomenclature	Mineral composition										
			Primary						Accessory		Secondary		
			Qz	Pl	Or	Mc	Bt	Ms	Aln	Zr	Chl	Sau	Ser
F1	Medium-grained (1–4 mm)	Biotite-gneissose granite	+	+	−	+	+	−	−	−	+	+	−
F2	Medium-grained (1–4 mm)	Perthite-gneissose granite	+	+	−	+	−	−	+	+	−	+	−
F3	Fine-grained (<1 mm)	Biotite-gneissose granite	+	+	−	+	+	−	−	−	+	+	−
F4	Coarse-grained (>5 mm)	Alkali-feldspar granite	+	+	+	−	−	+	−	−	−	−	−
F5	Medium-grained (3–5 mm)	Alkali-feldspar granite	+	+	+	+	+	−	−	−	+	+	+
F6	Medium-grained (3–5 mm)	Alkali-feldspar granite	+	+	+	+	+	−	−	−	+	+	+
F7	Medium-grained (3–5 mm)	Alkali-feldspar granite	+	+	+	+	+	−	−	−	+	+	+

*Qz* Quartz; *Pl* Plagioclase; *Or* Orthoclase; *Mc* Microcline; *Bt* Biotite; *Ms* Muscovite; *Aln* Allanite; *Zr* Zircon; *Chl* Chlorite; *Ser* Sericite, (+) presence,(−) absence

The petrographic description of the studied granites exhibited deformation effects expressed as fragmentation of quartz and plagioclase crystals. Moreover, the physico-mechanical properties may also be affected by the alteration processes, such as the saussuritization and chlorite formation as well as crystal deformation.

### Geochemistry

Twenty-one samples of seven plutonic rock types of Gabal El Faliq area were chemically analyzed for major oxides (%) and trace elements (ppm) and listed in Tables 2 and 3. From these tables, it is noticeable that the examined rocks exhibit a wide variation in their chemical compositions.

**Table 2:** Major oxides (%) of Gebel El-Faliq granitic rocks.

Rock name	No.	SiO_2_	Al_2_O_3_	TiO_2_	Fe_2_O_3_	MgO	Na_2_O	K_2_O	CaO	MnO	P_2_O_5_	SO_3_	LOI	Total
F1	F1-1	73.972	13.525	0.168	1.424	0.422	4.689	3.659	1.173	0.043	0.139	0.244	0.425	99.883
	F1-2	71.464	14.579	0.23	1.819	0.425	4.577	4.572	1.413	0.052	0.113	0.199	0.436	99.879
	F1-3	72.249	14.516	0.208	2.168	0.462	4.176	4.048	1.232	0.062	0.114	0.2	0.43	99.865
	**Av.**	**72.562**	**14.207**	**0.202**	**1.804**	**0.436**	**4.481**	**4.093**	**1.273**	**0.052**	**0.122**	**0.214**	**0.430**	**99.876**
F2	F2-1	77.734	11.854	0.052	1.52	0	2.95	4.863	0.407	0.019	0.023	0.03	0.444	99.896
	F2-2	75.104	12.794	0.071	1.946	0	2.881	6.083	0.494	0.023	0.019	0.025	0.431	99.871
	F2-3	75.838	12.727	0.064	2.312	0	2.623	5.371	0.428	0.028	0.019	0.025	0.47	99.905
	**Av.**	**76.225**	**12.458**	**0.062**	**1.926**	**0.000**	**2.818**	**5.439**	**0.443**	**0.023**	**0.020**	**0.027**	**0.448**	**99.891**
F3	F3-1	73.915	13.61	0.145	1.814	0.407	4.255	3.156	1.282	0.053	0.186	0.033	1.01	99.866
	F3-2	71.34	14.662	0.198	2.314	0.41	4.155	3.935	1.536	0.063	0.151	0.027	1.121	99.912
	F3-3	71.995	14.58	0.178	2.752	0.445	3.783	3.476	1.338	0.076	0.152	0.027	1.1	99.902
	**Av.**	**72.417**	**14.284**	**0.174**	**2.293**	**0.421**	**4.064**	**3.522**	**1.385**	**0.064**	**0.163**	**0.029**	**1.077**	**99.893**
F4	F4-1	74.745	13.24	0.109	1.505	0.181	4.254	3.809	0.79	0	0.059	0	1.11	99.802
	F4-2	72.232	14.277	0.149	1.923	0.182	4.156	4.76	0.952	0	0.047	0	1.21	99.888
	F4-3	72.976	14.208	0.135	2.29	0.198	3.788	4.21	0.829	0	0.048	0	1.14	99.822
	**Av.**	**73.318**	**13.908**	**0.131**	**1.906**	**0.187**	**4.066**	**4.260**	**0.857**	**0.000**	**0.051**	**0.000**	**1.153**	**99.837**
F5	F5-1	75.374	13.013	0.102	1.385	0	4.782	3.841	0.472	0.023	0.037	0.132	0.643	99.804
	F5-2	73.734	13.987	0.127	2.111	0	4.268	4.259	0.497	0.034	0.03	0.108	0.6	99.755
	F5-3	72.963	14.054	0.14	1.769	0	4.677	4.812	0.57	0.028	0.03	0.108	0.651	99.802
	**Av.**	**74.024**	**13.685**	**0.123**	**1.755**	**0.000**	**4.576**	**4.304**	**0.513**	**0.028**	**0.032**	**0.116**	**0.631**	**99.787**
F6	F6-1	74.701	13.296	0.077	1.499	0.184	5.136	3.451	0.391	0.019	0.057	0.029	0.991	99.831
	F6-2	73.045	14.284	0.096	2.283	0.201	4.583	3.824	0.411	0.027	0.047	0.024	1.01	99.835
	F6-3	72.34	14.361	0.106	1.913	0.186	5.025	4.324	0.471	0.022	0.046	0.024	1.07	99.888
	**Av.**	**73.362**	**13.980**	**0.093**	**1.898**	**0.190**	**4.915**	**3.866**	**0.424**	**0.023**	**0.050**	**0.026**	**1.024**	**99.851**
F7	F7-1	74.073	13.369	0.142	2.116	0.249	4.639	3.595	0.583	0.02	0.11	0	0.957	99.853
	F7-2	71.504	14.407	0.194	2.695	0.251	4.528	4.483	0.7	0.024	0.089	0	0.971	99.846
	F7-3	72.099	14.316	0.175	3.209	0.272	4.125	3.953	0.609	0.029	0.089	0	0.961	99.837
	**Av.**	**72.559**	**14.031**	**0.170**	**2.673**	**0.257**	**4.431**	**4.010**	**0.631**	**0.024**	**0.096**	**0.000**	**0.963**	**99.845**

Significant values are in bold.

**Table 3:** Trace elements abundance (ppm) of Gebel El-Faliq granitic rocks.

Rock name	No.	Sc	V	Cr	Co	Ni	Cu	Zn	Ga	As	Rb	Sr	Y	Zr	Nb	Sn	Ba	Hf	W	Pb	Th	U
F1	F1-1	4	4.3	5.9	20.7	3.3	4.2	40.7	20.8	10.4	122	164	8	125	12.5	22.1	311	5.2	467	11	19.2	1.5
	F1-2	2.4	5.3	5.3	19.7	1.8	3.8	39.6	19	8.4	119	161.5	7.8	123.4	11.6	3.3	292	2.3	438	7	16.7	1
	F1-3	3.2	4.8	5.6	20.2	2.55	4	40.15	19.9	9.4	120.5	162.7	7.9	124.2	12.05	12.7	301.5	3.75	452.5	9	17.95	1.25
	*Av.*	*3.2*	*4.8*	*5.6*	*20.2*	*2.6*	*4.0*	*40.2*	*19.9*	*9.4*	*120.5*	*162.7*	*7.9*	*124.2*	*12.1*	*12.7*	*301.5*	*3.8*	*452.5*	*9.0*	*18.0*	*1.3*
F2	F2-1	0.3	0.7	3	25.2	1.9	4	80.7	23.2	8.8	98.6	26	34.4	202	28.9	16.1	37	5.2	411	7.3	6.2	1.2
	F2-2	UDL	0.5	4.9	22	1.5	4.2	77	22	7.8	96.6	25.9	33	201.5	28	UDL	26.6	6.1	395	5.2	4.6	UDL
	F2-3	0.3	0.6	3.95	23.6	1.7	4.1	78.85	22.6	8.3	97.6	25.95	33.7	201.7	28.45	16.1	31.8	5.65	403	6.25	5.4	1.2
	Av.	0.3	0.6	4.0	23.6	1.7	4.1	78.9	22.6	8.3	97.6	26.0	33.7	201.7	28.5	16.1	31.8	5.7	403.0	6.3	5.4	1.2
F3	F3-1	3.5	5.7	4.2	16.3	1.1	4.5	46.7	22.1	11.4	128	157.4	10	162.5	15.3	18.4	303	7	389	11	17.9	0.9
	F3-2	2.4	2.3	5.6	14.7	2.1	4	47	20.5	12	126	155	10.5	161	15	UDL	288	6.6	367	8	14.6	1
	F3-3	2.95	4	4.9	15.5	1.6	4.25	46.85	21.3	11.7	127	156.2	10.25	161.7	15.15	18.4	295.5	6.8	378	9.5	16.25	0.95
	*Av.*	*3.0*	*4.0*	*4.9*	*15.5*	*1.6*	*4.3*	*46.9*	*21.3*	*11.7*	*127.0*	*156.2*	*10.3*	*161.7*	*15.2*	*18.4*	*295.5*	*6.8*	*378.0*	*9.5*	*16.3*	*1.0*
F4	F4-1	1.9	1.7	9.1	15.4	1.1	3.5	24.4	20.2	10	124	24.6	37	127	38	6.1	89.5	6.1	322	2.2	18.2	1.6
	F4-2	1.8	UDL	11.7	17.7	1.1	3.7	24.8	20	11.3	126.4	25	37.7	129.6	38.4	17.1	121.8	9.8	337	6	18.6	2
	F4-3	1.85	1.7	10.4	16.55	1.1	3.6	24.6	20.1	10.65	125.2	24.8	37.35	128.3	38.2	11.6	105.6	7.95	320.5	4.1	18.4	1.8
	*Av.*	*1.9*	*1.7*	*10.4*	*16.6*	*1.1*	*3.6*	*24.6*	*20.1*	*10.7*	*125.2*	*24.8*	*37.4*	*128.3*	*38.2*	*11.6*	*105.6*	*8.0*	*326.5*	*4.1*	*18.4*	*1.8*
F5	F5-1	4.1	UD	8	17.4	0.5	3.6	37	20.5	10	138	15.9	37.7	117	37.5	9.2	101	5.6	398	4.1	17.5	1.5
	F5-2	0.9	UDL	6.7	19	1.6	3.7	37.2	21	9.2	140.2	16.4	38.8	120.4	37.6	15.8	119.6	6.5	412	6	18	1.9
	F5-3	2.5	UDL	7.35	18.2	1.05	3.65	37.1	20.75	9.6	139.1	16.15	38.25	118.7	37.55	12.5	110.3	6.05	4505	5.05	17.75	1.7
	*Av.*	*2.5*	*UDL*	*7.4*	*18.2*	*1.1*	*3.7*	*37.1*	*20.8*	*9.6*	*139.1*	*16.2*	*38.3*	*118.7*	*37.6*	*12.5*	*110.3*	*6.1*	*420.2*	*5.1*	*17.8*	*1.7*
F6	F6-1	0.4	1.1	6.5	18.8	0.8	4.1	41.6	18.5	10	122.5	25.8	33	117	30.4	10	159.8	7.1	379	4	19.8	1.1
	F6-2	1.5	UDL	7.5	20.5	2	4.1	43.3	19.4	9.6	125.5	26.4	33.5	119	30.8	20.3	174.5	7.9	395	7.3	19.2	1.6
	F6-3	0.95	1.1	7	19.65	1.4	4.1	42.45	18.95	9.8	124	26.1	33.25	118	30.6	15.15	167.1	7.5	387	5.65	19.5	1.35
	*Av.*	*1.0*	*1.1*	*7.0*	*19.7*	*1.4*	*4.1*	*42.5*	*19.0*	*9.8*	*124.0*	*26.1*	*33.3*	*118.0*	*30.6*	*15.2*	*167.1*	*7.5*	*387.0*	*5.7*	*19.5*	*1.4*
F7	F7-1	3.5	1.4	15.8	17	0.6	4	65	19.8	8.9	123	28.7	33.5	129	38	2.4	118	8.7	314	5.5	17.5	1.8
	F7-2	1.4	3.2	12.6	18.2	1.4	4.1	65.3	20.4	10.3	127	29.7	34.5	132	38.6	19.7	142.5	8.8	328	9.4	17.7	1.4
	F7-3	2.45	2.3	14.2	17.6	1	4.05	65.15	20.1	9.6	125	29.2	34	130.5	38.3	11.05	130.2	8.75	321	7.45	17.6	1.6
	**Av.**	**2.5**	**2.3**	**14.2**	**17.6**	**1.0**	**4.1**	**65.2**	**20.1**	**9.6**	**125.0**	**29.2**	**34.0**	**130.5**	**38.3**	**11.1**	**130.2**	**8.8**	**321.0**	**7.5**	**17.6**	**1.6**

Significant values are in bold and italics.

The geochemical characteristics of Gabal El Faliq granitic intrusion were previously investigated^45^. This section is concerned with the geochemical composition of variable granitic rocks of Gabal El Faliq area. The major oxides and trace elements of the studied rock types were analyzed using XRF and the results are listed in Tables 2 and 3.

From Table 2**,** it is noticeable that the studied rocks show a wide variation in their chemical composition. The content of SiO_2_ varies from 71. 34 to 77.73 % (av. 73.49 %), Al_2_O_3_ ranges from 11.85 to 14.66 % (av. 13.79 %), the total Fe_2_O_3_^T^ ranges from 1.38 to 3.21 % (av. 2.04 %), and the total alkalis (K_2_O+Na_2_O) content ranges from 5.77 to 11.22 % (av. 8.5 %).

Regarding the granitic rock type (F2), it exhibited the highest silica content (av. 76.22), while the lowest alumina content (av. 12.45) relative to other rocks of the area under investigation. However, based on the petrographic examination, this rock type is classified as gneissose granite (due to the outer deformations). Moreover, based on its mineralogical component, this type is relatively fresh (plagioclase reveals saussuritizaion). Therefore, their chemical composition may be ascribed to highly feldspar fractionation.

**Figure 6:**
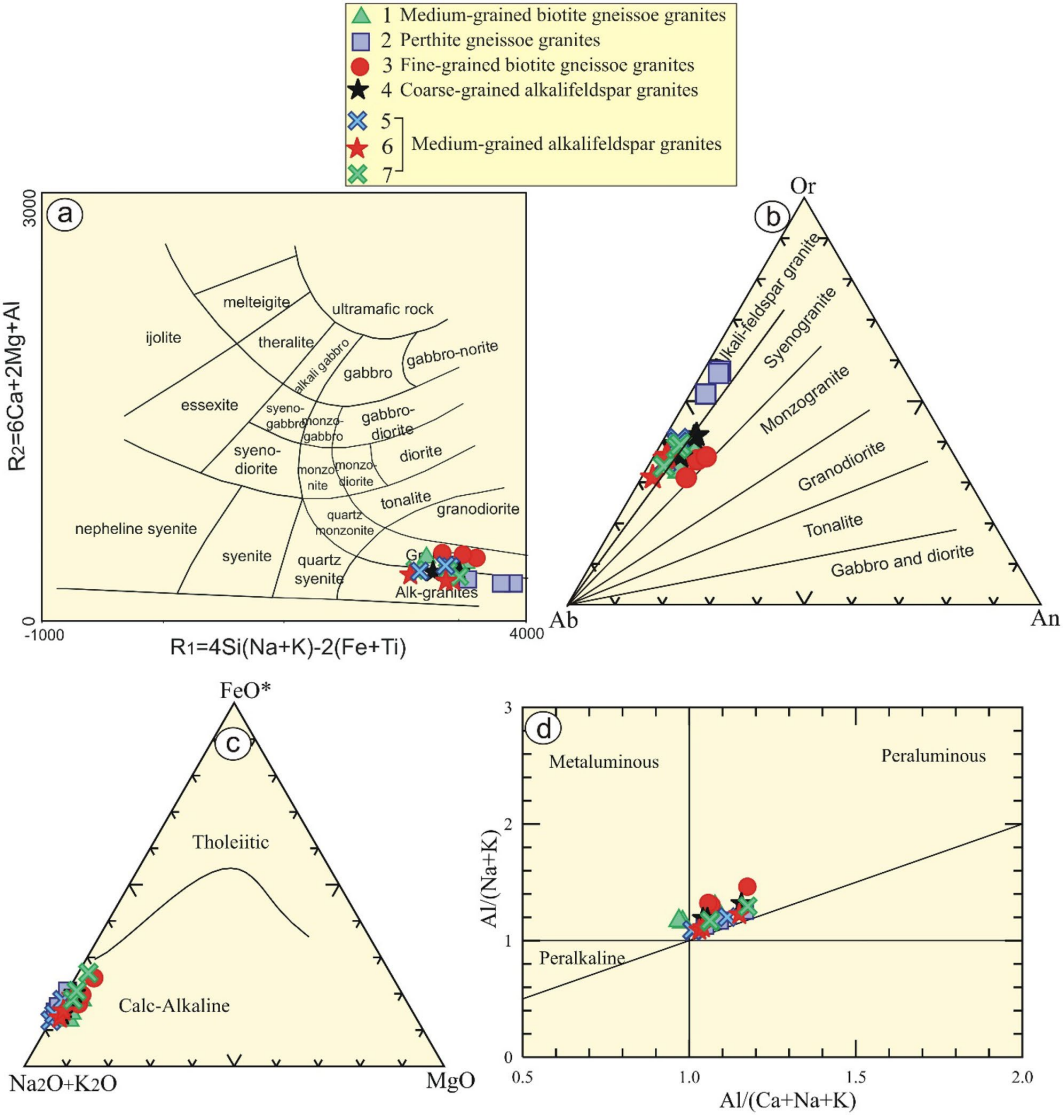
Geochemical diagrams of Gabal El Faliq granitic rocks: (**a**) R1-R2 diagram of (De la Roche et al., 1980)^55^; (**b**) Ab-Or-An normative diagram of (Streckeisen, 1976)^50^; (**c**) AFM ternary diagram of (Irvine and Baragar, 1971)^56^ and (**d**) ACNK vs. ANK binary diagram of (Shand, 1951)^57^.

Variable discrimination diagrams can be used to classify the studied granitic rocks of Gabal El Faliq area. In terms of R1-R2 diagram^55^, all rock samples (F2, F5, F6, and F7) locate in the alkali-granites field except samples (F1 and F3) and plotted in granite field (Fig. 6a). Further constraints, based on Ab-Or-An ternary diagram^50^, samples (F1 and F3) lie within syenogranite field, while others occupy alkali-feldspar granite field (Fig. 6b).

It is noticeable that the examined rock samples have a calc‐alkaline affinity (Fig. 6c) according to AFM (Na_2_O+K_2_O-Fe_2_O_3_^T^-MgO) diagram^56^. Further constrains, their calc‐alkaline signature is suggested by their agpaitic index (AI) < 0.87. Moreover, they revealed "peraluminous affinity" as indicated by alumina saturation index (ASI), where A/CNK> 1.1. This is supported by A/CNK- A/NK binary diagram^57^ (Fig. 6d).

**Figure 7:**
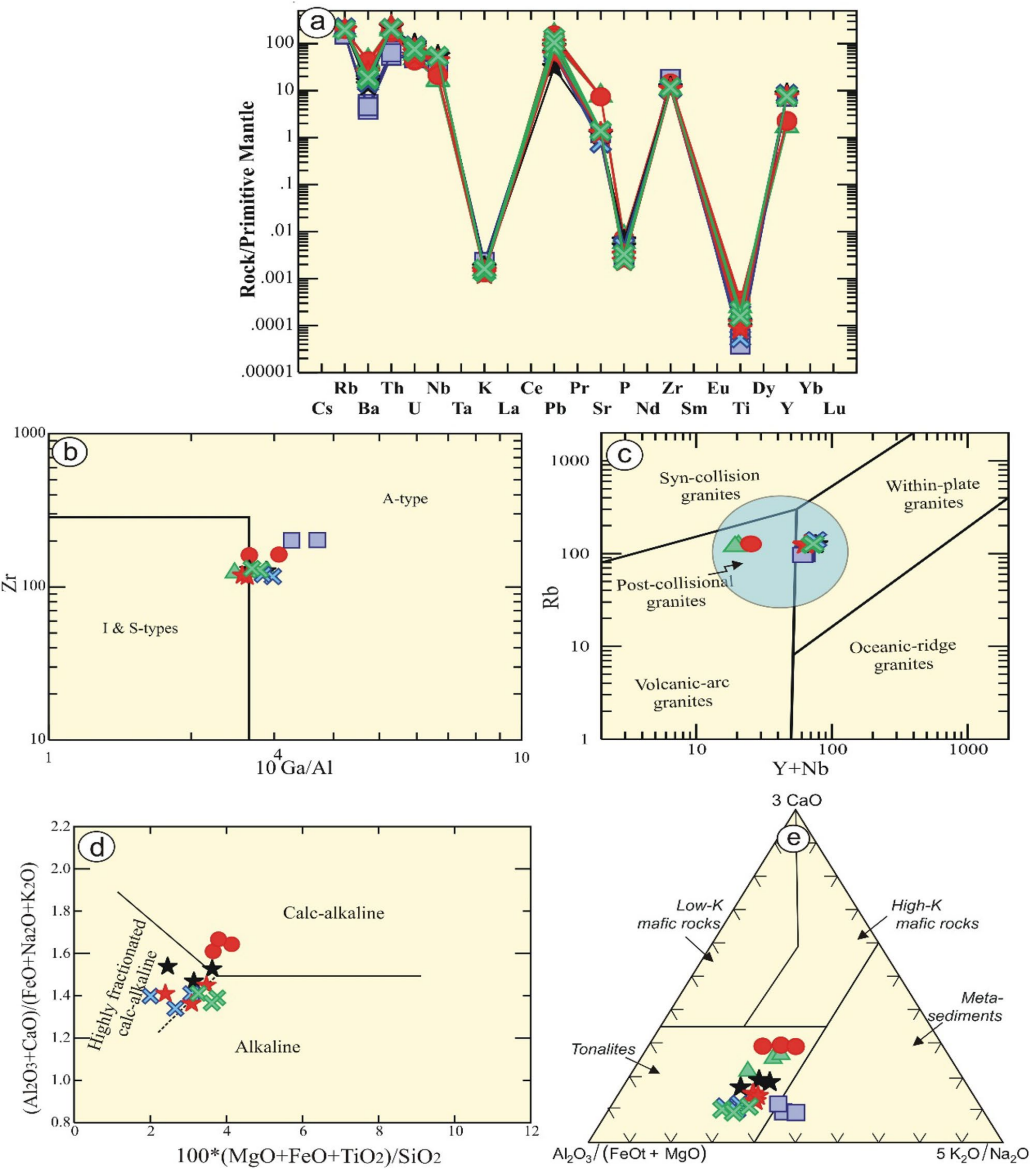
Geochemical diagrams of Gabal El Faliq granitic rocks: (**a**) Multi-trace elements normalized to primitive mantle by (Sun and McDonough 1989)^56,58^; (**b**) Zr vs. 104Ga/Al binary diagrams by (Whalen et al. 1987)^59^; (**c**) Nb + Y vs. Rb binary diagram by (Pearce et al. 1984)^60^; (**d**) Discrimination diagram by (Sylvester 1989)^61^ in which the rocks are > 68 wt. % SiO2; and (**e**) Source ternary diagram by (Laurent et al. 2014)^62^.

On the other hand, the examined samples of Gabal El Faliq granitic intrusion exhibit a wide variation from samples (F1 and F3) (syenogranites) that are enriched by biotite and plagioclase relative to other samples. It is observed that they are enriched with Sr (av. 159.5 ppm) and Ba (av. 298.5 ppm) relative to the average value (av. 24.44 ppm for Sr, and 109 for Ba) of the other samples (F 2, 4, 5, 6, and 7). Multi-trace elements are normalized to primitive mantle^58^, (Fig. 7a). They exhibit strong K, Ba, Sr, and Ti negative anomalies, reflecting highly fractional crystallization of feldspars, and titanite minerals. Controversy, they reveal positive Rb, Pb, Zr and Y anomalies.

### Tectonic emplacement

Its widely known that the granitic rocks are attributed to syn-orogenic (calc-alkaline), late- post-orogenic highly fractionated granitoids (calc-alkaline to alkaline) and post-orogenic (alkaline) granitic rocks^7,18^. The grey granite rocks represent the older ones and include rocks such as tonalite and granodiorite that are developed in volcanic arc setting, whereas the youngest rocks (alkaline) are commonly attributed to within plate / rift related granites^15,16,19,21^.

In the current research, the authors would like to link the mineralogical/chemical composition of the examined rocks and their tectonic regime with their geotechnical and engineering aspects. Therefore, by using the geochemical diagrams, all the examined granitic rocks are represented by highly evolved calc-alkaline granitic rocks (formed by fractional crystallization of I-type) of the post-orogenic regime.

The tectonic emplacement of the examined rocks can be manifested by using several geotectonic diagrams. The investigated rocks contain high content of Zr and Ga/Al ratio, reflecting an orogenic (A-type) granites^59^, with the exception of sample No. F1 that plot has I-type affinity (Fig. 7b). This result is supported by binary diagram^60^, where all samples plot in A-type field, whereas F1 and F3 samples plot in the field of volcanic arc granites due to less content of Y and Nb elements. In addition, all samples plot within post-collisional granites. In spite of, some of these samples plotted in the sector of A-type granites, have a geochemical characteristic of calc- alkaline granites (Fig. 7c), and highly fractionated calc-alkaline rocks^61^ (Fig. 7d). This is related to the extensive fractional crystallization of I-type (tonalite) melt^62^ (Fig. 7e).

### Normative mineral composition (NORM)

Based on the chemical analysis (oxides percentages) of the studied granitic rocks using XRF (X-Ray Fluorescence), the mineral composition of these rocks was calculated using "CIPW classification". Such classification was based upon the reorganizing of the chemical analysis from percentages of oxides into amounts of “standard minerals”^50,63^ as given in (Table 4). The hypothetical standard mineral composition of the studied granitic rock based upon "CIPW classification" revealed that the predominant mineralogical composition in all studied rocks is detected in the following order "Quartz: SiO_2_", "Albite: NaAlSi_3_O_8_", "Orthoclase: KAlSi_3_O_8_", "Anorthite: CaAl_2_Si_2_O_8_" along with some accessory minerals such as "Apatite: Ca_5_(Cl.F) (PO_4_)_3_", "Rutile: TiO_2_", "Corundum: Al_2_O_3_", in addition to iron-mineral group such as "Haematite: Fe_2_O_3_", "Ilmenite: FeTiO_3_".

**Table 4:** Normative mineral composition of Gebel El-Faliq granitic rocks.

Rock types	No.	Normative mineral composition										
		Or	Ab	An	Qz	Ap	Hem	Ilm	Hyp	Crn	Rt	Sum
F1:	F1-1	21.624	39.677	4.89	29.959	0.332	1.424	0.092	1.051	0.058	0.12	99.23
	F1-2	27.019	38.729	5.734	24.131	0.268	1.82	0.107	1.046	0	0.024	99.25
	F1-3	23.934	35.285	5.384	29.491	0.261	2.16	0.128	1.146	1.299	0.143	99.23
	*Av.*	*24.19*	*37.90*	*5.34*	*27.86*	*0.29*	*1.80*	*0.11*	*1.08*	*0.45*	*0.10*	*99.24*
F2:	F2-1	28.721	24.962	1.884	41.165	0.054	1.52	0.043	0	1.046	0.027	99.42
	F2-2	35.931	24.37	2.307	34.09	0.045	1.95	0.049	0	0.625	0.045	99.41
	F2-3	31.735	22.17	1.999	39.193	0.045	2.31	0.051	0	1.874	0.037	99.41
	*Av.*	*32.13*	*23.83*	*2.06*	*38.15*	*0.05*	*1.93*	*0.05*	*0.00*	*1.18*	*0.04*	*99.42*
F3:	F3-1	18.651	36.005	5.135	34.27	0.441	1.814	0.113	1.021	1.312	0.085	98.85
	F3-2	23.225	35.116	6.611	28.701	0.355	2.314	0.128	1.021	1.156	0.131	98.76
	F3-3	20.507	31.985	5.668	33.619	0.355	2.75	0.15	1.109	2.528	0.099	98.77
	*Av.*	*20.79*	*34.37*	*5.80*	*32.20*	*0.38*	*2.29*	*0.13*	*1.05*	*1.67*	*0.11*	*98.79*
F4:	F4-1	22.516	35.962	3.527	33.654	0.142	1.51	0	0.448	0.832	0.12	98.71
	F4-2	28.13	35.116	4.406	27.71	0.111	1.92	0	0.448	0.676	0.149	98.67
	F4-3	24.88	31.985	3.804	32.94	0.114	2.29	0	0.493	2.04	0.135	98.68
	*Av.*	*25.18*	*34.35*	*3.91*	*31.43*	*0.12*	*1.91*	*0.00*	*0.46*	*1.18*	*0.13*	*98.69*
F5:	F5-1	22.693	40.447	2.09	31.975	0.088	1.38	0.049	0	0.227	0.076	99.03
	F5-2	28.426	39.516	2.175	26.456	0.237	1.77	0.06	0	0.364	0.109	99.11
	F5-3	25.175	36.132	2.27	31.617	0.071	2.11	0.073	0	1.512	0.089	99.05
	*Av.*	*25.43*	*38.70*	*2.18*	*30.02*	*0.13*	*1.75*	*0.06*	*0.00*	*0.70*	*0.09*	*99.06*
F6:	F6-1	20.389	43.459	1.562	30.681	0.135	1.499	0.041	0.458	0.534	0.056	98.81
	F6-2	25.53	42.478	2.031	25.46	0.109	1.913	0.047	0.463	0.681	0.081	98.79
	F6-3	22.575	38.755	1.732	30.742	0.111	2.28	0.058	0.498	1.976	0.066	98.79
	**Av.**	**22.83**	**41.56**	**1.78**	**28.96**	**0.12**	**1.90**	**0.05**	**0.47**	**1.06**	**0.07**	**98.80**
F7:	F7-1	21.216	39.262	2.159	32.046	0.261	2.116	0.043	0.62	1.059	0.12	98.90
	F7-2	23.343	34.905	2.44	31.536	0.211	3.21	0.062	0.678	2.36	0.142	98.89
	F7-3	26.476	38.247	2.891	26.449	0.211	2.69	0.051	0.623	1.055	0.167	98.86
	**Av.**	**23.68**	**37.47**	**2.50**	**30.01**	**0.23**	**2.67**	**0.05**	**0.64**	**1.49**	**0.14**	**98.88**

Significant values are in bold and italics.

*Or* Orthoclase, *Ab* Albite, *An* Anorthite, *Qz* Quartz, *Ap* Apatite, *Hem* Hematite, *Ilm* Ilmenite, *Hyp* Hyperthene, *Crn* corundum, *Rt* Rutile.

These minerals were detected in different proportions from rock type to other. It was detected that the quartz content varied from (av. 27.86%) for (F1 rock type) to (av. 38.129%) for (F2 rock type). Regarding the predominant feldspar mineral content, it was detected that the "albite" is the main plagioclase minerals with an average content ranging from (23.83%) for (F2 rock type) to (41.56%) for (F6 rock type). The second predominant feldspar mineral is alkali-feldspar particularly "orthoclase" with an average content ranging from (20.79%) for (F3 rock type) to (32.129%) for (F2 rock type).

### Engineering properties (physical and mechanical)

The geotechnical assessment of granitic rocks in terms of engineering properties and thermal behavior are the main parameters used to evaluate their suitability as dimension or ornamental stones for the construction and building purposes^51^. According to ASTM specification, the requirements of granitic rocks for use as dimension stones including (water absorption, density, compressive strength, modulus of rupture, abrasion resistance, and flexural strength) were listed under the term “physical properties”.

**Figure 8:**
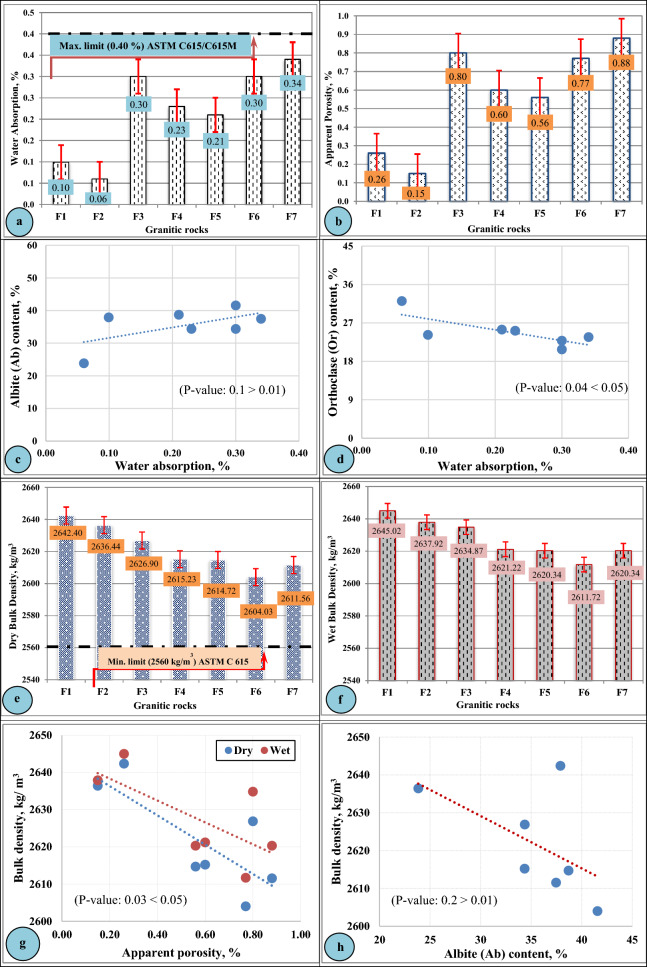

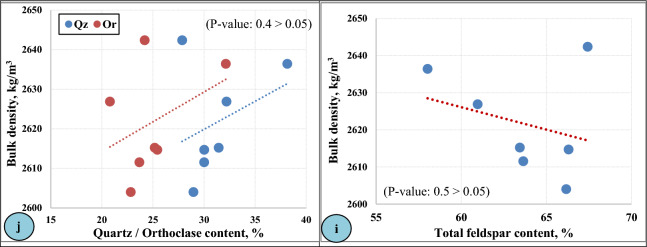
Physical properties of Gebel El-Faliq granitic rocks and their relationships with mineralogical composition.

**Figure 9:**
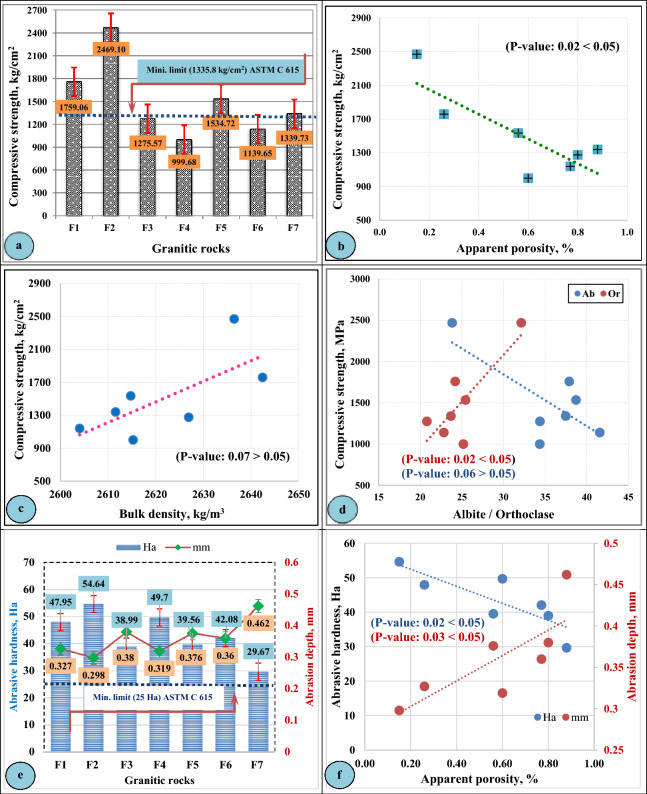
Mechanical properties of Gebel El-Faliq granitic rocks and their relationships with physical properties and mineralogical content.

In the present study, the engineering properties of the investigated granitic rocks at Gebel El-Faliq area were divided into two groups: (1) the physical properties group including (water absorption and bulk density in addition to apparent porosity); and (2) the mechanical properties group that including (compressive strength, abrasion resistance). These properties were graphically plotted and shown in (Figs. 8 and 9**)**.

#### Physical properties

Figure 8 illustrates the physical properties of the studied granitic rocks, and it was observed that the results of water absorption (Fig. 8a) ranged from 0.06% for (F2-rock type) to 0.34% for (F7-rock type) that were matching with the results of their apparent porosity (Fig. 8b) that ranged from 0.15% to 0.88%, respectively, which confirm the very strong positive relationship between apparent porosity and water absorption. The variation in these properties may be related to the variation in mineralogical composition as shown in (Fig. 8c and d). This figure shows that the water absorption behaves in line with albite content, while in the case of orthoclase, it has an inverse behavior.

The values of water absorption fall within the standard specification limits (< 0.40%)^51^. The current results were found parallel to the findings obtained by (Alzahrani et al. 2022^7^ and Rashwan et al. 2023^32^) on different types of granites that ranged from 0.14% to 0.52% for water absorption and from 0.36% to 1.36% for apparent porosity. On the contrary, other studies such as (Siegesmund et al. 2018^22^, Török and Török 2015^64^ and Freire-Lista et al. 2022^65^) investigated several types of igneous rocks and recorded high ranges of water absorption and apparent porosity values that ranged from 0.78% to 3.53% and from 0.3% to 6.66%; respectively.

The results of bulk density (dry and wet) of the studied granitic rocks were illustrated (Fig. 8e and f). It was observed from this figure that the values of dry and wet densities ranged from 2604.03 kg/m^3^ and 2611.72 kg/m^3^ (F 6—rock type) to 2642.4 kg/m^3^ and 2645.02 kg/m^3^ (F 1—rock type); respectively. These results matched in an inverse relationship with the results of the apparent porosity as shown in (Fig. 8g). Although the similarity between apparent porosity values of (F3 and F6—rock types), there is a difference in bulk density between them. Therefore, the variation in the values of rock densities was considered a function of their mineralogical content, where there is an inverse relationship between the albite content of rocks and their bulk density (Fig. 8h), while a positive relationship between both quartz/orthoclase contents of rocks and their bulk density was reported (Fig. 8i). As the albite is the main mineral composition forming the studied granitic rocks than orthoclase, there is an inverse relationship between total feldspar content and the bulk density (Fig. 8j).

The values of bulk density of the present study were looked similar to the findings obtained by (Alzahrani el al. 2022)^7^ in the range of 2582 to 2644 kg/m^3^, (Török and Török 2015)^64^ in the range of (2.58–2.68 g/cm^3^), (Rashwan et al. 2023)^32^ in the range of 2590 to 2748 kg/m^3^, (Dionísio et al. 2021)^66^ in the range of 2.48–2.63 g/cm^3^ and (Freire-Lista et al. 2022)^65^ 2461–2649 kg/m^3^.

Comparing the results of the bulk density with the standard specification relating to granite dimension stone according to (ASTM C615)^51^, it was observed that all investigated rocks satisfied the requirements (2560 kg/m^3^ as a minimum limit).

#### Mechanical properties

Compressive strength and abrasion resistance are the most important mechanical properties that evaluate the durability and soundness of the rocks intended to be used as dimension stones for building purposes.

The results of compressive strength and abrasion resistance of Gebel El-faliq granitic rocks were graphically illustrated (Fig. 9). It was observed that the values of compressive strength ranged from 98.03 MPa (999.68 kg/cm^2^) recorded for (F4-rock type) to 242.13 MPa (2469.10 kg/cm^2^) recorded for (F2-rock type) as shown in (Fig. 9a). The variation in the compressive strength results could be a function of several parameters such as apparent porosity (Fig. 9b), bulk density (Fig. 9c), and feldspar content (Fig. 9d). From these relationships, it was observed that the compressive strength decreases with increasing apparent porosity and plagioclase, it increases with increasing bulk density and alkali feldspar content.

The results of abrasion resistance as shown in (Fig. 9e) ranged from 29.67 Ha (0.462 mm abrasion depth) for (F7-rock type) to 54.64 Ha (0.298 mm abrasion depth) for (F2-rock type). As the porosity of a rock may weaken the cohesion between its grains and consequently lowers its durability, (Fig. 9f) shows a positive relationship between abrasion resistance values (Ha) of the studied rocks and their apparent porosities.

Comparing the results of compressive strength and abrasion resistance of the studied granitic rocks with the standard specification relating to granite dimension stone^51^, it was found that all rock types achieved the minimum abrasion resistance limit (25 Ha). In the case of compressive strength, the rock types (F1, F2, F5, and F7) satisfied the requirements of compressive strength limit (131 MPa as a minimum limit), while the remaining rock types (F3, F4, and F6) were fallen slightly under the minimum requirement of the same specification, however, they can be adequate for the light duty purposes such as interior use and for the exterior use such as building cladding. Furthermore, they can be used provided that they are evaluated in terms of thermal expansion, durability, elastic modulus, and permanent volume change^51^. The summary of the engineering properties of the studied granitic rock types at Gabal El-Faliq area was presented in (Table 5).

**Table 5:** Summary of physical and mechanical properties of Gebel El- Faliq granitic rocks.

Rock No.	Predominant grain size	Physical and mechanical properties							
		Physical properties				Mechanical properties			
		Water absorption, %	Apparent porosity, %	bulk density, kg/m^3^ Dry Wet		Compressive Strength, MPa Kg/cm^2^		Abrasion resistance, Ha mm	
F1	Medium-grained (1-4 mm)	0.10	0.26	2642.40	2645.02	172.50	1759.06	47.95	0.327
F2	Medium-grained (1-4 mm)	0.06	0.15	2636.44	2637.92	242.13	2469.10	54.64	0.298
F3	Fine-grained (<1 mm)	0.30	0.80	2626.90	2634.87	125.09	1275.57	38.99	0.380
F4	Coarse-grained (>5 mm)	0.23	0.60	2615.23	2621.22	98.03	999.68	49.70	0.319
F5	Medium-grained (3-5 mm)	0.21	0.56	2614.72	2620.34	150.50	1534.72	39.56	0.376
F6	Medium-grained (3-5 mm)	0.30	0.77	2604.03	2611.72	111.76	1139.65	42.08	0.36
F7	Medium-grained (3-5 mm)	0.34	0.88	2611.56	2620.34	131.38	1339.73	29.67	0.462
Standard Specification Limits^50^		0.40 Max.	N/A	2560 Min.	N/A	131 Min.	1335.8 Min.	25 Min.	N/A

### Thermal behavior (Coefficient of linear thermal expansion, α)

The thermal expansion phenomenon occurs under increasing temperature in all substances and in all forms of matter. It also includes the contraction of matter upon decreasing temperature. During this phenomenon, the shape, length, and volume of the substance change with changing temperature^67^. Therefore, the increase in linear dimensions, such as the length, of any material with temperature can be used to quantify its thermal expansion^68^.

As early mentioned, the thermal expansion as a result of heat transfer, is one of the deteriorative reasons for the rocks. Therefore, due to the badly heat conductivity of granitic rocks, the thermal action on the rock surface is more intense than in its interior, and a tension force develops causing the formation of cracks in the outer surface of the rock^23^.

The numerical formulae for calculating the coefficient of linear thermal expansion can be grouped into two broad categories; the first one is "temperature range-dependent expansion", while the second is "single temperature—dependent expansion.

The first category is defined as "the average or the mean coefficient of linear thermal expansion (**α**_**m**_) over a temperature range^68–71^ according to the following general equation:

6 $${\boldsymbol{\alpha }}_{{\varvec{m}}}=\frac{({{\varvec{L}}}_{2}-{{\varvec{L}}}_{1})/{{\varvec{L}}}_{{\varvec{o}}}}{{{\varvec{T}}}_{2}-{{\varvec{T}}}_{1}}=\left(\frac{1}{{{\varvec{L}}}_{{\varvec{o}}}}\right)\boldsymbol{*}\boldsymbol{ }\left(\frac{\Delta {\varvec{L}}}{\Delta {\varvec{T}}}\right)$$

Where, **α**_***m***_ is related to the slope of the chord between two points on the curve of length against temperature (Fig. 7)^68,69^, and so it represents the expansion over a particular temperature range from **T**_**1**_ to **T**_**2**_, Lo represents the initial specimen's length at temperature **T**_**o**_ (reference temperature) that expands to **L**_**1**_ at temperature **T**_**1**_ then to **L**_**2**_ at temperature **T**_**2**_, while **ΔL** is the change in specimen's length for the temperature change **ΔT**.

The second category is termed “true coefficient of linear thermal expansion (**α**_**T**_) that is related to the derivative (***dL/dT***) at a single temperature^68,69^, which can be defined according to the following equation:

7 $${\boldsymbol{\alpha }}_{{\varvec{T}}}=\frac{{\varvec{d}}{\varvec{L}}/{\varvec{L}}{\varvec{o}}}{{\varvec{d}}{\varvec{T}}}=\boldsymbol{ }\boldsymbol{ }\frac{1}{{\varvec{L}}{\varvec{o}}}\frac{{\varvec{d}}{\varvec{L}}}{{\varvec{d}}{\varvec{T}}}$$

**Figure 10:**
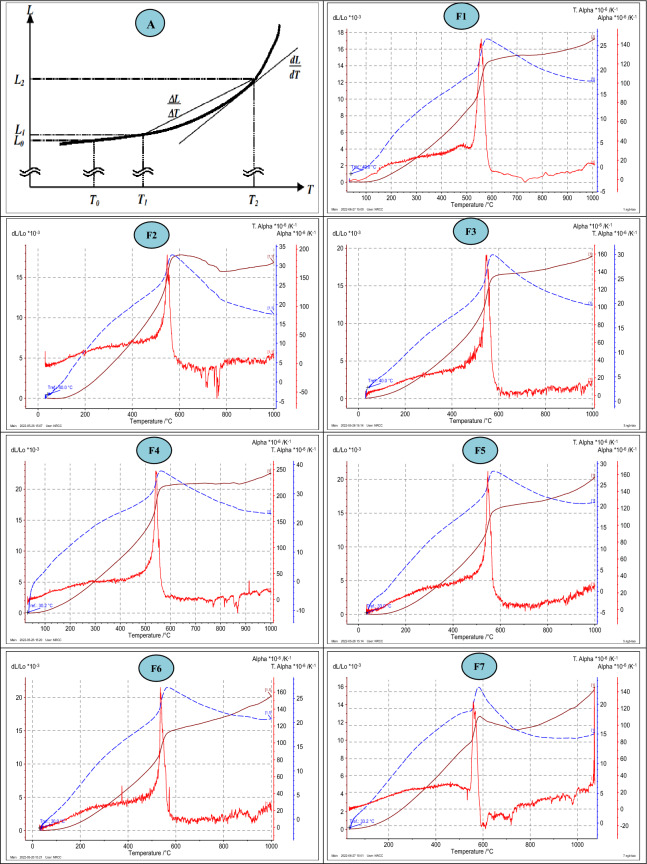
(A) Change in material length (L) as a function of temperature (T) (James et al. 2001)^68^; (F1–F7) change in coefficient of linear thermal expansion (α) and thermal strain of different intrusive granitic rocks under rising temperatures.

Where, **α**_**T**_ is the slope of the tangent to the curve of length against temperature as shown in (Fig. 10)^68^, dL/Lo is the derivative of thermal strain^72^. The results of the coefficient of linear thermal expansion (**α**) as well as the change in specimen's length as functions of temperature change for the studied intrusive granitic rock at Gebel El-Faliq area were graphically illustrated in (Fig. 10) and presented in Table 6.

**Table 6:** Average linear thermal expansion coefficient (α_m_, K^−1^) and length change (ΔL/Lo, %) or thermal strain of the studied rock samples.

Temperature, °C	Intrusive granitic rocks													
	Medium-grained (1–4 mm)				Fine-grained (< 1 mm)		Coarse-grained (> 5 mm)		Medium-grained (3–5 mm)					
	(F1)		(F2)		(F3)		(F4)		(F5)		(F6)		(F7)	
	α_m_ (x10^−6^), K^-1^	ΔL/Lo, %	α_m_ (x10^−6^), K^-1^	ΔL/Lo, %	αm (x10^−6^), K^-1^	ΔL/Lo, %	α_m_ (x10^−6^), K^-1^	ΔL/Lo, %	α_m_ (x10^−6^), K^-1^	ΔL/Lo, %	α_m_ (x10^−6^), K^-1^	ΔL/Lo, %	α_m_ (x10^−6^), K^-1^	ΔL/Lo, %
100	**0.772**	**0.0052**	**0.300**	**0.0021**	**5.531**	**0.0385**	**4.472**	**0.0311**	**0.658**	**0.0044**	**2.484**	**0.0173**	**3.744**	**0.0250**
200	6.085	0.1014	7.780	0.1317	9.402	0.1591	12.651	0.2154	6.346	0.1063	7.480	0.1267	7.656	0.1275
300	11.096	0.2966	14.463	0.3905	13.729	0.3704	19.360	0.5227	12.203	0.3262	13.143	0.3549	12.306	0.3289
400	14.885	0.5463	19.273	0.7125	17.129	0.6328	23.824	0.8809	16.648	0.6111	17.139	0.6336	15.766	0.5787
500	18.455	0.8614	23.798	1.1172	21.052	0.9876	28.358	1.334	20.985	0.9796	20.770	0.9750	18.489	0.8631
~ 573	**26.193**	**1.4395**	**32.316**	**1.742**	**29.570**	**1.6068**	**37.962**	**2.0215**	**28.413**	**1.5243**	**27.412**	**1.4622**	**23.009**	**1.2603**
600	25.826	1.4631	31.256	1.779	28.881	1.6454	36.217	2.0649	27.865	1.5814	26.637	1.5186	21.997	1.2463
700	22.841	1.524	25.569	1.7127	25.009	1.674	31.209	2.0906	24.626	1.6433	23.969	1.6055	17.189	1.147
800	20.018	1.5353	20.474	1.5756	22.369	1.7202	27.158	2.0901	22.191	1.7022	22.166	1.7058	14.947	1.1465
900	18.399	1.5947	18.649	1.6211	20.657	1.7964	24.233	2.1089	20.913	1.8128	21.304	1.8519	14.393	1.2476
1000	17.669	1.7077	17.388	1.6849	19.511	1.8911	23.271	2.2551	20.946	2.0246	20.843	2.0197	14.362	1.3882

Significant values are in bold.

Figure 10 shows the change in specimen's length ***(dL)***, as a function of thermal strain ***(dL/Lo, %),*** as well as the thermal coefficient (**α**_**m**_**, α**_**T**_) under the influence of the temperature increasing (***T***) up to 1000 °C. From this figure, it can observe an increase in the specimen's length with a gradual heating of the rocks over the all temperature range, while the thermal coefficient just increased up to (~573 °C) and returned to decrease over this temperature degree. There was a variation in the values of both change in specimen's length ***(dL)*** and mean thermal coefficient (**α**_**m**_) over the temperature range between the investigated rocks as given in (Table 6), such variation may be influenced by several parameters as follows:

#### Linear thermal expansion as a function of mineralogical composition

**Figure 11:**
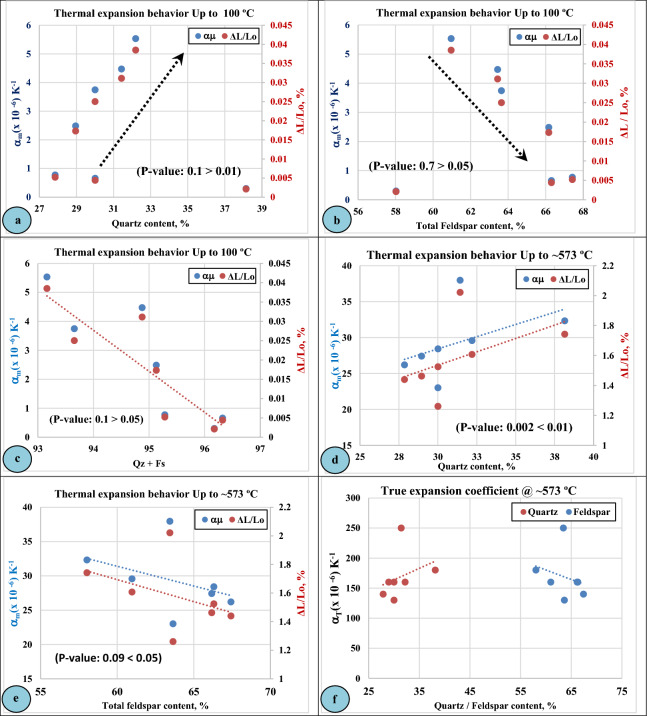
Linear Thermal Expansion (LTE) of Gabal El-Faliq granitic rocks as a function of mineralogical composition (quartz, feldspar content).

As it was known that the intrusive igneous rocks are of multi-mineralogical composition and the main mineral composition is quartz and feldspar (two types) in addition to minor minerals in different proportions. At 100 °C, the increase in quartz content led to an increase in the specimen's length and expansion coefficient (Fig. 11a), while the increase in total feldspar content (Fig. 11b) and the combination of quartz and feldspar content (Fig. 11c) resulted in a reduction in the length of the specimens along with expansion coefficient. This is due to the high thermal coefficient value of quartz mineral (around 16.66 × 10^−6^/°C) relative to that of feldspar group (4.16 × 10^−6^/°C for plagioclase and 3.68 × 10^−6^/°C for alkali feldspar)^67^. As given in (Table 6**)**, the minimum values of linear expansion along with expansion coefficient were recorded for (F2- Perthite-gneissose granites of medium-grained crystals) with (0.0021%) and (0.30 × 10^-6^/K^−1^), while the maximum values were recorded for (F3- Biotite-gneissose granites of fine-grained crystals) with (0.0385%) and (5.531 × 10^-6^ /K^−1^), respectively.

Correspondingly, the effect of quartz and total feldspar contents on the thermal behavior of the studied rocks at elevated temperatures demonstrated a similarity to that at lower temperatures. For example, at ~573 °C, the increase in quartz and feldspar content led to an increase and decrease in linear expansion and expansion coefficient (Fig. 11d–f), respectively.

As listed in (Table 6), the values of change in specimen length of the investigated rocks along with their thermal expansion coefficients up to (~573 °C) ranged from (1.2603%) and (23.009 × 10^−6^ / K^-1^) for (F7-Alkali-feldspar granite of a medium-grained size) to (2.0215 %) and (37.962 × 10^−6^ / K^−1^) for (F4- Alkali-feldspar granite of a coarse-grained size). Relating the thermal behavior of the investigated rocks, (up to ~573 °C), to their mineralogical composition, there was a positive relationship with quartz content (Fig. 11d), while a negative relationship with total feldspar content (Fig. 11e).

The influence of the mineralogical composition (Quartz and feldspar) of the studied granitic rocks on their thermal behavior was found similar to the findings reported by (Siegesmund et al. 2018^22^, De Castro and Paraguass 2004^23^).

Regarding the effect of higher temperatures, exceeding (~573 °C), on the thermal behavior of the studied rocks, a noticeable reduction in the thermal coefficient (**α**_**m**_) values was observed. This may be attributed to the phase transition of quartz mineral from (α to β) that appears at 573 °C^7^ and may influence the physical properties of rocks^72^. Such reduction seemed similar to the findings obtained by (Alzahrani et al. 2022)^7^. According to (Plevova et al. 2016)^25^ the rocks of high quartz content demonstrated a lower thermal expansion coefficient upon heating above ~ 573 °C (α-β quartz phase transition).

Regarding the thermal expansion coefficients at specific temperatures namely (true thermal expansion coefficient, **α**_**T**_), (Fig. 10) illustrates only one sharp peak (increase) for all studied granitic rocks at around 573 °C. The thermal coefficient values at this temperature ranged from (140 x 10^−6^ / K^−1^) for (F-1 Biotite-gneissose granites with medium-grained size) to (250 x 10^−6^ / K^−1^) for (F-4 Alkali-feldspar granites with coarse-grained size). Such variation in the *α*_*T*_ values may be attributed to the variation in the amounts of quartz and feldspar minerals between the studied granitic rocks as presented in (Table 4). Figure 11f illustrates the relationship between quartz and feldspar content in the studied granitic rocks and the intensity of thermal. The sudden increase in the thermal expansion coefficient at around 573 °C may be related to the transitional state of quartz mineral from α-quartz to β quartz phase^7,69^.

#### Linear thermal expansion as a function of chemical composition

**Figure 12:**
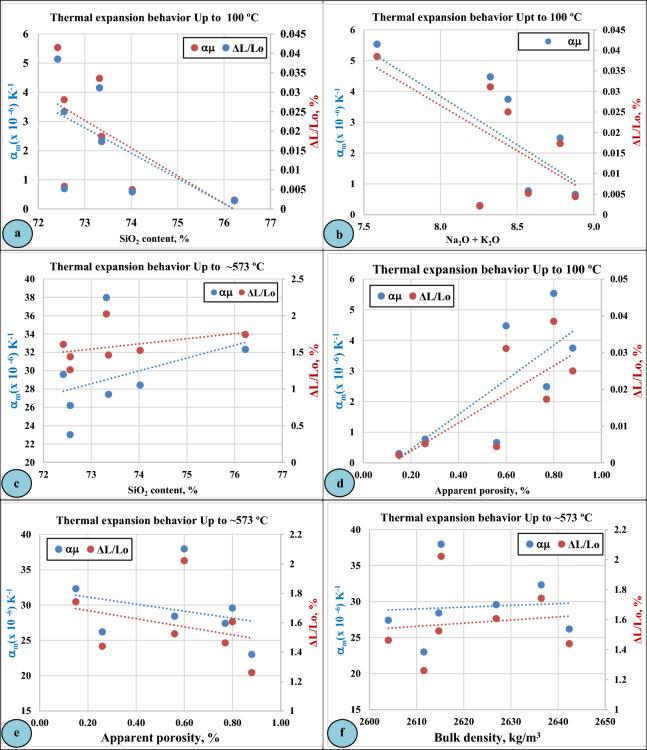
Linear Thermal Expansion (LTE) of Gabal El-Faliq granitic rocks as a function of chemical composition (SiO2, Na2O+K2O) and physical properties (apparent porosity, bulk density).

Similar to the mineralogical composition, the intrusive granitic rocks are characterized by a high content of silicon oxide (SiO_2_) that ranges in this study from (72.417–76.225%) in addition to fluxing or alkali oxides (Na_2_O and K_2_O). In the present study, the relationship between the main chemical composition of the studied granitic rocks represented by (SiO_2_ and Na_2_O + K_2_O) and their thermal behavior at low and high temperatures was illustrated in (Fig. 12).

Regarding the effect of the chemical composition of the studied intrusive rocks on their expansion behavior, it was observed that the rock samples of high (SiO_2_, %) and (Na_2_O+K_2_O, %), achieved lower values of linear thermal expansion than that of lower contents at lower temperatures (100 °C) (Fig. 12a,b).

On the contrary, the effect of (SiO_2_, %) content on the thermal behavior of the studied rocks at elevated temperatures (~ 573 °C) was different, where there is a positive relationship between the SiO_2_ content of rock samples and their thermal expansion values (Fig. 12c).

#### Linear thermal expansion as a function of physical properties

Figure 12d–f illustrates the linear thermal expansion behavior of different types of granitic rocks at Gebel El-Faliq area as a function of their physical properties such as apparent porosity and bulk density. According to (Siegesmund et al. 2018)^22^, micro cracks can avoid or enhance mineral expansion depending on their size, density, and orientation, therefore, they could play an important role in the thermal expansion. According to (De Castro and Paraguass 2004)^23^ the increase in apparent porosity and grain size of minerals caused a decrease in the thermal expansion of some Brazilian granitic rocks.

In the present study it can observe a positive relationship between the apparent porosity of the granitic rocks and their thermal behavior at lower temperatures (up to 100 °C), while at higher temperatures (up to ~ 573 °C) a negative relationship is observed. On the contrary, a positive relationship can be noticed between the bulk density of the studied rocks and their linear thermal expansion at higher temperatures (up to ~ 573 °C).

## Conclusions

The geotechnical assessment of any rock for use as dimension or ornamental stones in several building and structural purposes requires several geological and geotechnical studies. In the present study, the geological studies in terms of petrography, mineralogy, and geochemistry, along with the geotechnical measurements or engineering properties in terms of physical, mechanical, and thermal behaviors, were applied on different types of Neoproterozoic igneous rocks named "granite" on a commercial base, from Gebel El-Faliq area, Central Eastern Desert, Egypt for determining their suitability for using as dimension stones in different decorative purposes. The findings of geological and geotechnical studies were drawn as follows:

Based on the petrographic investigation, the studied intrusive rocks were classified into two main categories: *gneissose granites* (Biotite–Perthite) of medium- to fine-grained size and *alkali-feldspar granites* of coarse- to medium-grained size. Furthermore, some alteration minerals such as chlorite, saussurite, and sericite minerals were also petrographically detected.

The geochemical studies of the studied rock types revealed that the main mineral composition is albite, orthoclase and quartz in varying proportions, along with some accessory minerals such as apatite and rutile in addition to some minor quantities of iron-group minerals such as hematite and ilmenite.

The geotechnical measurements revealed that the maximum water absorption and apparent porosity values are 0.34% and 0.77%, respectively, while the minimum bulk density is 2604.03 kg/m^3^. The compressive strength results ranged from 999.68 to 2469.10 kg/cm^2^, while that of abrasion resistance varied from 29.67 to 54.64 Ha. Linking the petrographic analysis of rocks with their engineering performance, it is observed that the mineral composition has a remarkable effect on the physical and mechanical properties. The increase in albite content led to an increase in water absorption while a decrease in bulk density and compressive strength. This effect is reversed in case of orthoclase content. Furthermore, the increase in the grain size of rock leads to an increase in apparent porosity and decrease in compressive strength and abrasion resistance consequently.

The studied rock types exhibited great variations in their thermal expansion under changes in temperatures, mineral composition, and physical properties. The stepwise heating increased the thermal expansion coefficient until around 573 °C. The increase in quartz content led to an increase in expansion coefficient. On the contrary, the increase in feldspar content led to a decrease in the expansion coefficient. The increase in apparent porosity caused an increase in thermal expansion up to 100 °C, where the maximum change in rock length did not exceed 0.038%, which confirms the suitability of using the granitic rocks in outdoor decorative purposes (cladding/paving) under variable temperature conditions.

By comparing the results of the engineering properties with the limit of a standard specification related to dimension stones of granite, it was found that the requirements of the studied granitic rocks as a dimension stones were achieved.

### Recommendations

Based on the obtained results, it is highly recommended to apply these types of granitic igneous rocks as building flooring for interior purposes of light duty or as building cladding for exterior use.

### Future scope

The sizes and orientations (direction) of the mineral crystals, in addition to the mineral alterations in the igneous rocks should be studied intensively. These parameters are considered important parameters that may have a considerable role in the properties of rocks under temperature variations and loads.

## Data Availability

All data and materials availability statement is present within the text of the manuscript.
